# DNA barcoding and species delimitation of Chaitophorinae (Hemiptera, Aphididae)

**DOI:** 10.3897/zookeys.656.11440

**Published:** 2017-02-14

**Authors:** Xi-Chao Zhu, Jing Chen, Rui Chen, Li-Yun Jiang, Ge-Xia Qiao

**Affiliations:** 1 Key Laboratory of Zoological Systematics and Evolution, Institute of Zoology, Chinese Academy of Sciences, No. 1-5 Beichen West Road, Chaoyang District, Beijing 100101, P.R. China; 2 College of Life Science, University of Chinese Academy of Sciences, Shijingshan District, Beijing 100049, P.R. China

**Keywords:** Chaitophorinae, distance-based analysis, gnd, mitochondrial genes, tree-based analysis

## Abstract

Chaitophorinae aphids are widespread across Eurasia and North America, and include some important agricultural and horticultural pests. So, accurate rapid species identification is very important. Here, we used three mitochondrial genes and one endosymbiont gene to calculate and analyze the genetic distances within different datasets. For species delimitation, two distance-based methods were employed, threshold with NJ (neighbor-joining) and ABGD (Automatic Barcode Gap Discovery), and two tree-based approaches, GMYC (General Mixed Yule Coalescent) and PTP (Poisson Tree Process). The genetic interspecific divergence was clearly larger than the intraspecific divergence for four molecular markers. COI and COII genes were found to be more suitable for Chaitophorinae DNA barcoding. For species delimitation, at least one distance-based method combined with one tree-based method would be preferable. Based on the data for *Chaitophorus saliniger* and *Laingia psammae*, DNA barcoding may also reveal geographical variation.

## Introduction

Aphids from more than 5,000 species (Favret 2016) feed on plant phloem directly and spread various plant diseases (Blackman and Eastop 2000), many serving as important economic pests. The identification of aphid species based on morphological characteristics faces tremendous challenges due to their complicated life cycle, polymorphism, phenotypic plasticity, and numerous morphs (Zhang and Zhong 1983, Foottit et al. 2009a). Chaitophorinae lies within Aphididae, and comprises two tribes, Chaitophorini and Siphini, including 196 species and subspecies in 12 genera (Remaudiere and Remaudiere 1997, Favret 2016). The subfamily is distributed mainly in the Palaearctic (about 80% of species), and Nearctic (Richards 1972, Qiao 1996, Liu et al. 2009, Wieczorek 2010). Most species in this subfamily are monoecious holocyclic, but some species, such as Sipha (Sipha) flava and Sipha (Rungsia) maydis, may be anholocyclic in regions with milder winters (Blackman and Eastop 2000, Wieczorek 2010, Wieczorek and Bugaj-Nawrocka 2014). The Chaitophorini is mainly associated with plants of the families Salicaceae and Aceraceae (Blackman and Eastop 1994), whereas the Siphini infest plants in the Poaceae, Cyperaceae, Juncaceae and Typhaceae (Blackman and Eastop 2006). Additionally, individual species often have high host specificity (Blackman and Eastop 1994). Species identification of Chaitophorinae aphids can be difficult when based on their morphological characteristics. The Chaitophorini have clear morphological differences between genera, but *Chaitophorus* (109 known species) and *Periphyllus* (49 known species) have high species diversity (Essig 1952, Hille Ris Lambers 1960, Pintera 1987, Qiao 1996); and the morphological differences between species within these genera are relatively slight, often depending on the chaetotaxy of the body dorsum and appendages (Pintera 1987). In the Siphini, both among genera and between species, overlap and convergence of morphological characteristics are common, and genus and species identification are not easy. *Sipha* in particular (11 known species) has relatively great diversity, and species identification can be a problem.

DNA barcoding based on a short fragment of mitochondrial DNA can provide an effective tool for species diagnosis. In animals, the 5’end of mitochondrial cytochrome c oxidase I (COI) with a 658-bp fragment was selected as a standard DNA barcode (Hebert et al. 2003). This has been widely used for identifying unknown specimens and the rapid identification of species (Hebert et al. 2003, Wang and Qiao 2009, Wang et al. 2013b, Wen et al. 2013). Its practicability and effectiveness have been recognized and accepted in some insect groups, such as Diptera (Scheffer et al. 2006), Lepidoptera (Hajibabaei et al. 2006), Ephemeroptera (Ball et al. 2005), Hemiptera (Lee et al. 2011), Coleoptera (Lobl and Leschen 2005), and Hymenoptera (Smith et al. 2008). Additionally, the application range was expanded to pest control and quarantine (Armstrong and Ball 2005, Ratnasingham and Hebert 2007, Naaum et al. 2012, Pelletier et al. 2012). For aphids, the DNA barcoding approach has played an efficient role in the rapid identification of species on specific plants (Naaum et al. 2012, Chen et al. 2013b, Wang et al. 2013a, Wang et al. 2013b, Wen et al. 2013, Wang et al. 2015), the effective distinction of morphologically indistinguishable species and subspecies (Wang and Qiao 2009, Rebijith et al. 2013, Cocuzza and Cavalieri 2014, Beji et al. 2015, Kinyanjui et al. 2016), the recognition of cryptic species (Rebijith et al. 2013, Lee et al. 2015), and in species classification (Cocuzza and Cavalieri 2014, Mroz et al. 2015). Likewise, DNA barcoding may be used in species diversity assessment within different regions (Podmore et al. 2015, Chen et al. 2016); and is a powerful tool for the identification of multi-life stages, different morphs, and biological debris (Shufran and Puterka 2011). Crucially, it has improved the monitoring and control of pest aphids. The identification of aphid species is often difficult due to the shortage of easily distinguishable morphological characteristics, or feature convergence (Wang and Qiao 2009). Some chaitophorine species are important agricultural, forestry, and horticulture pests, for which accurate identification is necessary. At the authors’ last count (2016.04.06), researchers have provided some chaitophorine DNA barcoding sequences for 36 species to the NCBI and for 49 species to the Barcode of Life Data System (BOLD) (Foottit et al. 2008, Foottit et al. 2009b, Lee et al. 2011, Gwiazdowski et al. 2015). However, the DNA barcoding of this group is insufficient. In this work, we sequenced 1,609 sequences from 670 samples in 8 genera from both tribes of Chaitophorinae, based on three genes from the aphid mitochondrial genome, and one from the endosymbiont *Buchnera*. We employed four methods (threshold with NJ, ABGD, GMYC, and PTP) to analyze sequence diversities and genetic divergences between different species and probe the efficiency of identifying species. Based on DNA barcoding data, we also discuss the influence of geographical distribution on population differentiation.

## Materials and methods

### Taxa sampling and gene selection

All samples were collected into and cryopreserved in 95% or 100% ethanol. DNA from one individual per sample was isolated for molecular studies and three to five individual aphids per collection were mounted on microscope slides for morphological examination. Preserved aphid colonies were examined prior to preparation to ensure that they did not consist of multiple species. Voucher specimens for each sample were identified by G.X. Qiao based on morphological diagnostic features using standard literature-based keys (esp. Blackman and Eastop 1994, Pintera 1987, Wieczorek 2010) and by a comparison with previously identified specimens in the National Zoological Museum of China, Beijing. To avoid mutual influence and to ensure the independence of the different research methods, the morphological identification and molecular research were performed independently. All samples and voucher specimens were deposited in the National Zoological Museum of China, Institute of Zoology, Chinese Academy of Sciences, Beijing, China. Details of the sequenced taxa and voucher information are listed in Suppl. material 1.

Three aphid genes were targeted: mitochondrial cytochrome oxidase c subunit I (COI), cytochrome oxidase c subunit II (COII), and cytochrome b (Cytb), and one aphid endosymbiont *Buchnera* gene gluconate-6-phosphate dehydrogenase (gnd) (Kim and Lee 2008, Wang and Qiao 2009, Zhang et al. 2011, Chen et al. 2012).

### DNA extraction, amplification and sequencing

Total genomic DNA was extracted from single aphid. Individual aphids were selected from the ethanol-preserved candidates with a destructive DNA extraction procedure. Plump adults are the ideal experimental material, but they must be examined under a microscope (Leica DM 2500) to eliminate parasitized individuals. Total DNA was extracted by following the Quick-Start protocol of DNeasy Blood & Tissue Kit (QIAGEN, Dusseldorf, Germany) with a single individual. The DNA solution was then stored at -20 °C for subsequent molecular experiments.

The polymerase chain reaction (PCR) mixture for the amplification of COI, COII, Cytb, and gnd genes comprised 22 μl of double distilled water (ddH_2_O), 3 μl of 10 ×EasyTaq Buffer (+ Mg^2+^) (TransGen Biotech, Beijing, China), 2.4 μl of 2.5 mM/800 μl dNTPs (TransGen Biotech), 0.6 μl of 10 pmol/μl forward and reverse primers, 0.4 μl of 5 U/μl EasyTaq DNA Polymerase (TransGen Biotech), and 1 μl of DNA solution for a total volume of 30 μl.

The PCR conditions differed according to the gene and the specific primers, especially the annealing temperature, which was the most critical factor influencing product quality. The detailed primer information is shown in Suppl. material 2. The thermal setup of primer LepF/LepR (Foottit et al. 2008) or LCO1490/HCO2198 (Folmer et al. 1994) for COI gene fragment was: a 5-minute initial denaturation at 95 °C followed by 35 cycles of 30-second denaturation at 95 °C, 30 seconds of annealing at 50 °C, a 1-minute extension at 72 °C, and a 10-minute final extension at 72 °C. The protocol for primer mt2993+ (Stern 1994)/A3772 (Normark 1996) for tRNA/COII molecular marker was as follows: a 5-minute initial denaturation at 95 °C followed by 35 cycles of 1-minute denaturation at 95 °C, 1 min at 42 °C , 1 min at 72 °C, and a 7-minute final extension at 72 °C. The parameters of primer CP1/CP2 (Harry et al. 1998) for Cytb amplification was simplified as: 94 °C with 5 min, and 40 cycles of 94 °C with 50s, 48 °C with 1 min, 72 °C with 1.5 min, and 72 °C with 10 min. The setup of primer BamHI/ApaI (Clark et al. 1999) for *Buchnera* gnd gene was predigested as: 94 °C with 5 min, and 30 cycles of 94 °C with 1 min, 55 °C with 30s, 72 °C with 1 min, and 72 °C of 10 min final extension.

The amplification products were detected by 1.5% agarose gel electrophoresis (AGE), and then purified using EasyPure Quick Gel Extraction Kit (TransGen Biotech). The eligible products were then sent to TsingKe Biological Technology, Beijing, China or BGI, Shenzhen, China for sequencing, which was required to be bidirectional.

### Sequence edition and alignment

The returned forward and reverse chromatograms were loaded and then assembled and edited by SeqMan in DNAStar software (DNASTAR, Madison, Wisconsin, USA). The nucleotide sequences were first examined in NCBI by Basic Local Alignment Search Tool (BLAST) (Altschul et al. 1990) to test their affiliations. Concurrently, for the encoding gene fragments, we translated the assembled contigs into amino acids by MEGA6 (Tamura et al. 2013) to examine whether the sequences were correct and accurate. Multiple alignments were accomplished by MAFFT (Katoh and Standley 2013), and the sequences were then adjusted and trimmed manually in MEGA6. It is noteworthy that the sequences amplified with primer mt2993+/A3772 covered the COII gene fragment as well as a tRNA, which was then removed for subsequent analysis.

### Species delimitation methods

In addition to sequences from 425 samples, we downloaded 245 COI and 1 COII sequence from NCBI. Here, we defined the datasets as COI-670 (including the whole research group and NCBI sequences), COII-376 (including 375 internal sequences and 1 NCBI sequence), Cytb-413 (newly gotten for this study), gnd-396 (newly obtained sequences), and COI-338, COII-338, Cytb-338, gnd-338, which contained only the specimens that acquired all 4 gene sequences.

A neighbor-joining (NJ) (Saitou and Nei 1987) tree was constructed by MEGA6 based on the aligned sequences. One thousand bootstrap replications were calculated to assess the credibility of the NJ analysis. The Kimura 2-parameter (K2P) model of base substitution (Kimura 1980) was selected in pairwise distances calculation, and for the more accurate comparison between sequences, the pairwise deletion pattern was selected for gaps/missing data treatment. After bootstrap consensus trees with bootstrap values at each node were obtained, we computed the condensed tree with a 50% cut-off value for the consensus tree. When analyzing the COI-670 tree, we chose a threshold of 2% (Foottit et al. 2009b) for a cluster standard, which has been well used in aphids. With regard to the COII-376, COII-338, Cytb-413, Cytb-338, gnd-396 and gnd-338 NJ trees, we calculated only the cluster topologies.

The Automatic Barcode Gap Discovery (ABGD) (Puillandre et al. 2012a) approach is a model-based method for delimiting species. Based on the existence of a barcoding gap (namely the intraspecific divergences are smaller than interspecific divergences) and a prior intraspecific divergence (*p*), the ABGD procedure first sorts the dataset into a hypothetical species, and then computes recursively with the previous groups to obtain a result optimized until there are no better partitions. We ran the ABGD with a graphic web version (http://wwwabi.snv.jussieu.fr/public/abgd/abgdweb.html). First, we calculated the distance values among samples by using MEGA6 with p-distance, Jukes-Cantor (JC69) model, and K2P model separately, and the result data were saved as CSV format file. We then chose 0.055, which had been suggested for Aphididae (Foottit et al. 2009b), as the prior intraspecific divergence for COI-670 and COI-338 datasets, and we used a *p* value of 0.1, which was the default and shown to be sufficient for analysis, for the other data sets. The other parameters were maintained by default for all analyses.

The General Mixed Yule Coalescent (GMYC) (Fujisawa and Barraclough 2013) is a tree-based approach for the delimitation of species. We ran the GMYC method in R project (available from: https://www.r-project.org/) by using the “splits” package (available from: http://r-forge.r-project.org/projects/splits). The input tree was required to be strictly ultrametric and bifurcating, which meant there was no zero-length branch. Here, we used a maximum likelihood (ML) tree as the input. Therefore, the haplotypes were calculated and generated by DnaSP (Librado and Rozas 2009), and an ML tree was constructed by RAxML (Stamatakis 2006) with haplotype data. Due to the ultrametric and bifurcating requirement, the ML tree was constructed with r8s (Sanderson 2003). The outcome tree modified by r8s was read into the “splits” R package, and the delimiting result was obtained with relevant commands.

The Poisson Tree Process (PTP) (Zhang et al. 2013) model is another tree-based method for inferring putative species. The PTP approach is a close relative of the GMYC method, but it only needs a simple phylogenetic tree as its input without requiring it to be ultrametric and bifurcating. As an updated version of the original PTP, the bPTP method was employed simultaneously to separate hypothetical species, which added a Bayesian support value to the tree. The PTP and bPTP analyses were run on a web server (http://species.h-its.org/ptp/) and the value 500,000 was selected for MCMC generations, with the other parameters set by default. The input tree was an ML tree constructed by RAxML with GTRCAT model. However, we encountered the same problem as Schwarzfeld and Sperling (2015), namely that the bPTP analysis failed to show convergence under 500,000 generations (the upper limit of the web server). Therefore, only the PTP result is displayed and discussed below.

## Results

### Morphological identification

The 425 samples collected by the group members in recent years were carefully authenticated with mounted individuals under the microscope, and all 425 samples were identified to species. The few vouchers with uncertain species identification were sorted into featured clusters and were given the epithet “sp.”, which made them convenient for further analysis. A total of 75 morphological species were determined from 670 whole samples, and 51 were identified from the 425 mounted samples.

### Sequence alignment

The COI sequences were trimmed to a length of 658 bp, which included 365 conserved sites, 293 variable sites and 258 parsimony-informative sites. The sequences had an average nucleotide composition of 38.0% T, 17.1% C, 34.4% A, and 10.5% G. The COII sequences were trimmed to a final length of 672 bp, among which 399 sites were conserved, 273 sites were variable, and 251 sites were parsimony-informative. The average T, C, A, G compositions of these sequences were 38.7%, 14.0%, 39.5%, and 7.8%, respectively. The Cytb gene was 760 bp, in which there were 420 conserved sites, 340 variable sites and 303 parsimony-informative sites. The Cytb sequences consisted of 41.4% T, 15.3% C, 34.3% A, and 9.0% G. We obtained a total length of 807 bp for the gnd gene with an average nucleotide composition of 37.8% T, 9.8% C, 39.5% A, and 12.8% G, among which there were 368 conserved sites, 439 variable sites and 417 parsimony-informative sites. Across all 4 genes, a strong T and A nucleotide composition bias existed.

From a total of 425 samples, 425 COI gene fragment sequences, 375 COII gene fragment sequences, 413 Cytb gene fragment sequences, and 396 gnd gene fragment sequences were acquired. The successive amplification efficiency of those markers in order was COI (100%) > Cytb (97%) > gnd (93%) > COII (88%).

### Genetic divergence analysis

Genetic divergences were assessed by 5 disparate metrics among and within species. For the interspecific divergences of congeneric species, we chose the average interspecific distance, which was calculated within genera that contained more than one species, and the smallest interspecific distance, which meant the minimal value of interspecific distance within genera with at least two species. When evaluating the intraspecific divergences, three variables (average intraspecific distance, mean theta, and average coalescent depth) were applied. The average intraspecific distance was the average value of the genetic distances between samples within species that had at least two individuals. The mean theta signified a modified theta, which expressed the average pairwise distance scored for species with more than one obtained representative, by dislodging improper individuals concerned with the asymmetrical acquisition of samples. The average coalescent depth, namely the average value of maximum intraspecific distance, was calculated for species in which there were no fewer than two samples.

All five interspecific and intraspecific metrics were determined within genera and species (Table 1). The results of different genes and datasets showed distinctly high interspecific divergences and low intraspecific distances. For DNA barcoding the smallest interspecific distance and average coalescent depth were the most useful and intuitive parameters. Within Chaitophorinae, the genetic divergence ranges of smallest interspecific distance and average coalescent depth of COI, COII, Cytb, and gnd were (0.0693–0.1233, 0.0060–0.0218), (0.0563–0.1110, 0.0024–0.0070), (0.0703–0.1060, 0.0052–0.0230), and (0.0807–0.1427, 0.0010–0.0090), respectively. The figures above were obtained across the whole dataset. To obtain a more reliable and comparable analysis, we calculated the results of the 338-sample datasets. The smallest interspecific distance and average coalescent depth ranges of COI-338, COII-338, Cytb-338, and gnd-338 were (0.0693–0.0995, 0.0044–0.0165), (0.0563–0.1190, 0.0024–0.0090), (0.0703–0.1040, 0.0052–0.0230), and (0.0811–0.1600, 0.0010–0.0090), respectively. The computations above showed a properly high smallest interspecific distance and a comparatively low average coalescent depth.

**Table 1.** The inter- and intra-specific genetic distances of congeneric species of Chaitophorinae.

	Interspecific Distance		Intraspecific Distance		
Genus/Dataset (no. species/specimens)	average interspecific distance	smallest interspecific distance	average intraspecific distance	mean theta	average coalescent depth
***Chaitophorus***					
**COI-670(38/534)**	0.1158±0.0191	0.1015±0.0178	0.0070±0.0060	0.0083±0.0057	0.0126±0.0126
**COII-376(25/283)**	0.0956±0.0246	0.0853±0.0207	0.0017±0.0019	0.0025±0.0019	0.0060±0.0054
**Cytb-413(25/323)**	0.1233±0.0281	0.0971±0.0260	0.0049±0.0066	0.0058±0.0068	0.0219±0.0357
**gnd-396(25/306)**	0.0996±0.0316	0.0807±0.0248	0.0020±0.0030	0.0034±0.0032	0.0042±0.0051
**COI-338(25/253)**	0.1117±0.0286	0.0995±0.0215	0.0058±0.0044	0.0077±0.0033	0.0088±0.0071
**COII-338(25/253)**	0.0950±0.0247	0.0855±0.0208	0.0018±0.0021	0.0027±0.0021	0.0062±0.0055
**Cytb-338(25/253)**	0.1169±0.0310	0.0983±0.0270	0.0043±0.0064	0.0052±0.0067	0.0164±0.0337
**gnd-338(25/253)**	0.0843±0.0264	0.0811±0.0255	0.0014±0.0016	0.0025±0.0014	0.0032±0.0033
***Lambersaphis***					
**COI-670(1/3)**	-	-	0.0040±0.0028	0.0060±0.0000	0.0060±0.0000
**COII-376(1/3)**	-	-	0.0047±0.0033	0.0070±0.0000	0.0070±0.0000
**Cytb-413(1/3)**	-	-	0.0053±0.0012	0.0053±0.0012	0.0070±0.0000
**gnd-396(1/3)**	-	-	0.0007±0.0005	0.0010±0.0000	0.0010±0.0000
**COI-338(1/3)**	-	-	0.0040±0.0028	0.0060±0.0000	0.0060±0.0000
**COII-338(1/3)**	-	-	0.0047±0.0033	0.0070±0.0000	0.0070±0.0000
**Cytb-338(1/3)**	-	-	0.0053±0.0012	0.0053±0.0012	0.0070±0.0000
**gnd-338(1/3)**	-	-	0.0007±0.0005	0.0010±0.0000	0.0010±0.0000
***Periphyllus***					
**COI-670(19/83)**	0.1113±0.0231	0.1075±0.0220	0.0040±0.0146	0.0080±0.0198	0.0218±0.0439
**COII-376(13/53)**	0.0936±0.0299	0.0938±0.0282	0.0007±0.0014	0.0027±0.0015	0.0024±0.0024
**Cytb-413(14/54)**	0.0975±0.0200	0.0944±0.0194	0.0020±0.0029	0.0041±0.0030	0.0052±0.0032
**gnd-396(14/54)**	0.1256±0.0669	0.1292±0.0602	0.0004±0.0010	0.0016±0.0014	0.0007±0.0012
**COI-338(13/53)**	0.0971±0.0258	0.0985±0.0248	0.0019±0.0032	0.0056±0.0032	0.0044±0.0035
**COII-338(13/53)**	0.0936±0.0299	0.0938±0.0281	0.0007±0.0014	0.0027±0.0015	0.0024±0.0024
**Cytb-338(13/53)**	0.0974±0.0203	0.0935±0.0206	0.0020±0.0029	0.0041±0.0030	0.0052±0.0032
**gnd-338(13/53)**	0.1250±0.0679	0.1283±0.0632	0.0004±0.0010	0.0016±0.0014	0.0007±0.0012
***Trichaitophorus***					
**COI-670(3/3)**	0.1233±0.0200	0.1233±0.0200	-	-	-
**COII-376(3/3)**	0.1103±0.0190	0.1103±0.0190	-	-	-
**Cytb-413(2/2)**	0.1040±0.0000	0.1040±0.0000	-	-	-
**gnd-396(3/3)**	0.1427±0.0162	0.1427±0.0162	-	-	-
**COI-338(2/2)**	0.0990±0.0000	0.0990±0.0000	-	-	-
**COII-338(2/2)**	0.1190±0.0000	0.1190±0.0000	-	-	-
**Cytb-338(2/2)**	0.1040±0.0000	0.1040±0.0000	-	-	-
**gnd-338(2/2)**	0.1600±0.0000	0.1600±0.0000	-	-	-
***Yamatochaitophorus***					
**COI-670(3/3)**	0.0043±0.0009	0.0043±0.0009	-	-	-
**COII-376(3/3)**	0.0037±0.0021	0.0037±0.0021	-	-	-
**gnd-396(3/3)**	0.0007±0.0005	0.0007±0.0005	-	-	-
***Chaetosiphella***					
**COI-670(3/24)**	0.0515±0.0418	0.0693±0.0490	0.0149±0.0128	0.0197±0.0111	0.0185±0.0165
**COII-376(3/24)**	0.0372±0.0368	0.0563±0.0399	0.0083±0.0051	0.0091±0.0047	0.0090±0.0090
**Cytb-413(3/24)**	0.0481±0.0455	0.0703±0.0498	0.0140±0.0123	0.0154±0.0120	0.0230±0.0220
**gnd-396(3/23)**	0.0521±0.0608	0.0887±0.0627	0.0084±0.0068	0.0107±0.0058	0.0090±0.0090
**COI-338(3/23)**	0.0512±0.0422	0.0693±0.0490	0.0147±0.0128	0.0202±0.0107	0.0165±0.0145
**COII-338(3/23)**	0.0371±0.0369	0.0563±0.0399	0.0083±0.0052	0.0091±0.0048	0.0090±0.0090
**Cytb-338(3/23)**	0.0480±0.0457	0.0703±0.0498	0.0142±0.0124	0.0158±0.0121	0.0230±0.0220
**gnd-338(3/23)**	0.0521±0.0608	0.0887±0.0627	0.0084±0.0068	0.0107±0.0058	0.0090±0.0090
***Laingia***					
**COI-670(1/2)**	-	-	0.0640±0.0000	0.0640±0.0000	0.0640±0.0000
**COII-376(1/2)**	-	-	0.0680±0.0000	0.0680±0.0000	0.0680±0.0000
**Cytb-413(1/2)**	-	-	0.0620±0.0000	0.0620±0.0000	0.0620±0.0000
***Sipha***					
**COI-670(5/17)**	0.0940±0.0250	0.0882±0.0320	0.0082±0.0127	0.0147±0.0139	0.0118±0.0159
**COII-376(2/5)**	0.1115±0.0009	0.1110±0.0000	0.0027±0.0012	0.0027±0.0012	0.0040±0.0000
**Cytb-413(2/5)**	0.1073±0.0013	0.1060±0.0000	0.0048±0.0031	0.0058±0.0024	0.0090±0.0000
**gnd-396(1/4)**	-	-	0.0005±0.0005	0.0010±0.0000	0.0010±0.0000
**COI-338(1/4)**	-	-	0.0033±0.0021	0.0040±0.0017	0.0060±0.0000
**COII-338(1/4)**	-	-	0.0027±0.0012	0.0027±0.0012	0.0040±0.0000
**Cytb-338(1/4)**	-	-	0.0048±0.0031	0.0058±0.0024	0.0090±0.0000
**gnd-338(1/4)**	-	-	0.0005±0.0005	0.0010±0.0000	0.0010±0.0000

Notes: Interspecific divergences were calculated using the average interspecific distance and smallest interspecific distance. Intraspecific divergences were evaluated by the average intraspecific distance, mean theta, and average coalescent depth. The average interspecific distance was calculated within genera that contained more than one species. The smallest interspecific distance was defined as the minimal value of interspecific distance within genera with at least two species, the average intraspecific distance was the average value of the genetic distances between samples within those species that had at least two individuals, the mean theta was expressed as the average pairwise distance scored from species with more than one obtained representatives by dislodging improper individuals concerned with the asymmetric procurement of samples, and the average coalescent depth was the average value of maximum intraspecific distances.

To observe the occurrence frequency of different genetic divergences, we drew the frequency line charts of inter- and intra-specific genetic distances based on 338 datasets (Figure 1). Each gene was signified in one chart: the top half was calculated with all 338 samples; and the bottom half was scored by eliminating two samples (Nos. 25138 and 25161) of *Chaetosiphella longirostris*. The overlap region was indicated by the red dotted rectangle. No obvious barcoding gap was found in these samples across COI, COII, Cytb, and gnd genes. The overlap regions of COI, COII, Cytb, and gnd in the top half were 0.000–0.031, 0.000–0.023, 0.000–0.045, 0.000–0.018; and those in the bottom half were 0.022–0.031, 0.011–0.023, 0.015–0.045, 0.006–0.018, respectively. It was clear that the overlap region was much narrower by eliminating the questioned vouchers, and the total frequency within that region was also reduced significantly (Figure 1). The data in the overlap region represented samples that the DNA barcoding would fail to identify. Therefore, a lower total frequency in that region was better. The order of the total frequencies in the overlap region was COI < COII < Cytb < gnd in the top and bottom half parts.

**Figure 1.**
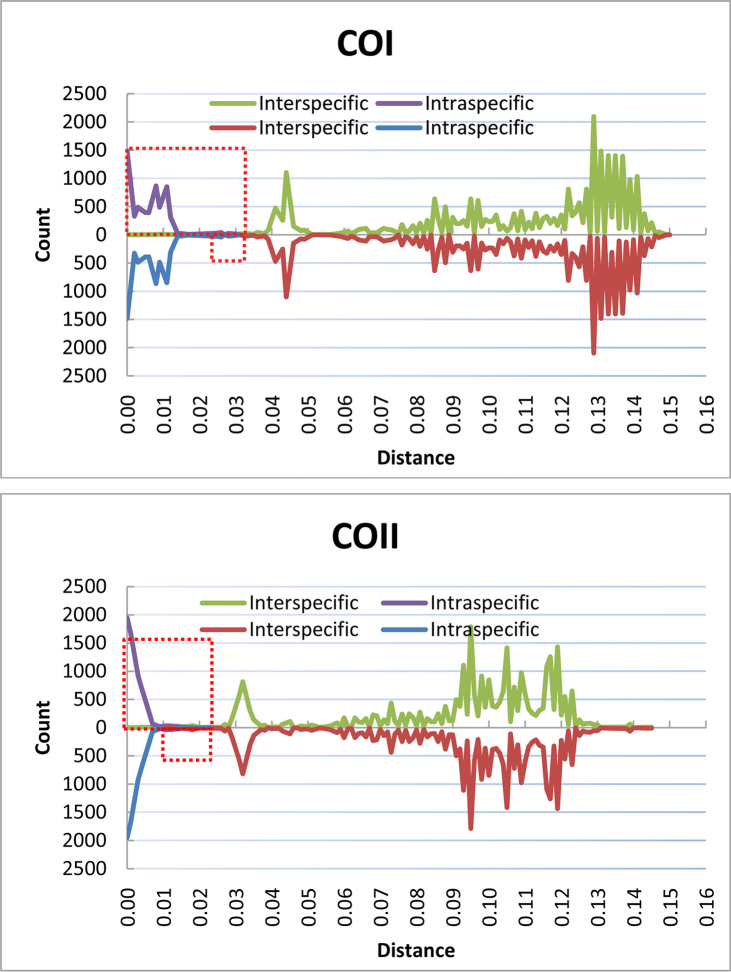
Frequency line charts of inter- and intra-specific genetic distances based on 338 dataset. The x-axis represents the genetic distance, and the y-axis represents the occurrence times in the whole genetic distance matrix. Each peak was a data point with corresponding genetic distance and occurrence times. The data points on the green and red line were calculated with the interspecific distances, and the points on purple and blue line were calculated with the intraspecific distances. The overlap region, which was the crossing area of inter- and intra-specific divergence, is indicated by the red dotted rectangle. Each gene was signified in one chart: the top half was calculated with all the 338 samples; and the bottom half was scored by eliminating the queried samples of *Chaetosiphella longirostris*.

**Figure 1.**
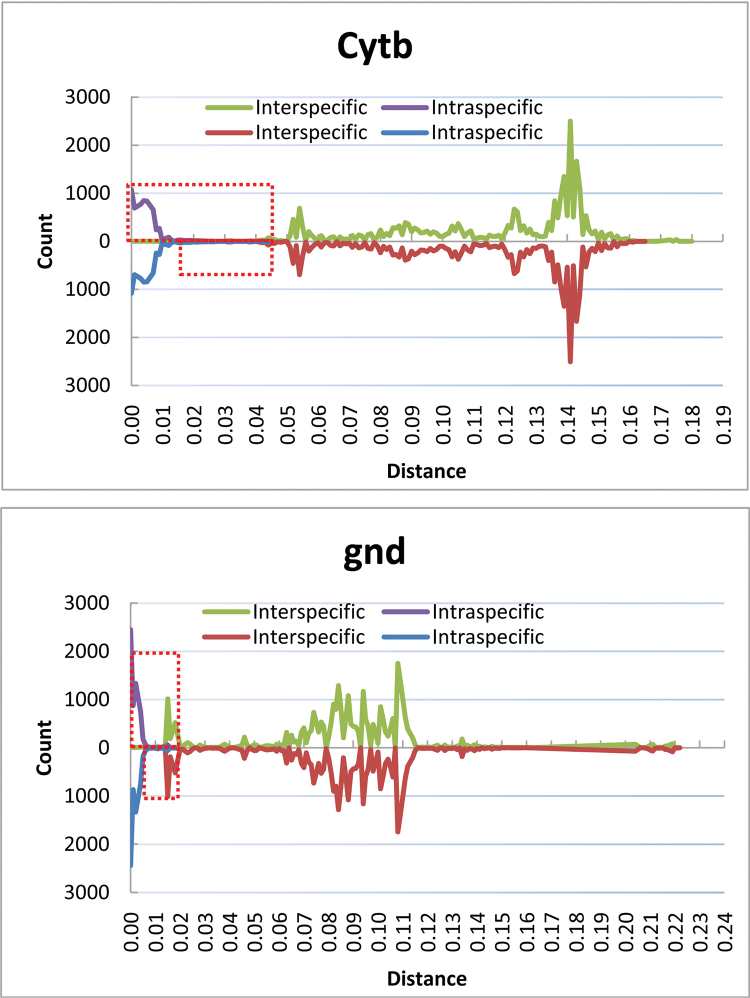
Continue.

### Species delimitation

Given that there were no unambiguous and credible references of genetic thresholds for COII, Cytb, and gnd in aphids, the method of threshold with NJ was applied only in the COI-670 dataset with a threshold of 2% (Foottit et al. 2009b). For all the COI, COII, Cytb and gnd datasets, one distance-based method ABGD (for the analysis results, see Suppl. material 3 for details) and two tree-based approaches GMYC and (b)PTP were employed simultaneously as reference counterpoints to each other.

The morphological and molecular identification results are shown in Table 2. To suitably display the analysis results, four group names were used: *accurate*, of which the putative species clusters were identical with morphological identifications; *split*, whose component vouchers were merely part of a specific unabridged morphological species without representatives of others; *lumped*, which was defined as an aggregation of more than one species including all samples of those species; and partial lumped, a multi-species cluster that consisted of all the vouchers of one or several species as well as part samples of other species.

**Table 2.** Analysis results of different datasets with various approaches.

Dataset/Method	Morphology	Cluster number	Accurate	Split	Lumped	Partial lumped
**COI-670**	75					
**GMYC**		89	65.17%	31.46%	2.25%	1.12%
**PTP**		85	67.06%	28.24%	3.53%	1.18%
**ABGD**		81	72.84%	23.46%	2.47%	1.23%
**threshold with NJ**		81	72.84%	23.46%	2.47%	1.23%
**COII-376**	51					1.89%
**GMYC**		53	83.02%	13.21%	1.89%	
**PTP**		48	83.33%	8.33%	8.33%	0.00%
**ABGD**		50	82.00%	12.00%	6.00%	0.00%
**Cytb-413**	48					
**GMYC**		54	79.63%	18.52%	0.00%	1.85%
**PTP**		49	81.63%	12.24%	2.04%	4.08%
**ABGD**		48	79.17%	12.50%	4.17%	4.17%
**gnd-396**	49					
**GMYC**		46	86.96%	4.35%	8.70%	0.00%
**PTP**		45	84.44%	4.44%	11.11%	0.00%
**ABGD**		48	87.50%	6.25%	4.17%	2.08%
**COI-338**	45					
**GMYC**		47	89.36%	8.51%	0.00%	2.13%
**PTP**		49	85.71%	12.24%	0.00%	2.04%
**ABGD**		46	91.30%	6.52%	0.00%	2.17%
**COII-338**	45					
**GMYC**		45	93.33%	4.44%	2.22%	0.00%
**PTP**		45	86.67%	8.89%	4.44%	0.00%
**ABGD**		45	86.67%	8.89%	4.44%	0.00%
**Cytb-338**	45					
**GMYC**		50	80.00%	18.00%	0.00%	2.00%
**PTP**		46	80.43%	13.04%	2.17%	4.35%
**ABGD**		42	83.33%	4.76%	7.14%	4.76%
**gnd-338**	45					
**GMYC**		42	92.86%	0.00%	7.14%	0.00%
**PTP**		41	90.24%	0.00%	9.76%	0.00%
**ABGD**		44	93.18%	2.27%	2.27%	2.27%

Notes: The morphology column gives the number of morphological species of different datasets. Cluster number represents the putative species amount of each method. Accurate represents which putative species clusters were identical with morphological identifications; and split represents which component vouchers were merely part of a specific unabridged morphological species without representatives of others. Lumped, was defined as an aggregation of more than one species and the entire samples of those species were included, whereas partial lumped was defined as a multi-species cluster, which consisted of all the vouchers of one or several species as well as part samples of other species.

Seventy-five morphological species were identified from COI-670, which included sequences downloaded from NCBI. For COI-670, we obtained 89 putative species by the GMYC approach with a 65.17% accuracy rate, 85 species using PTP with 67.06% accuracy, 81 species using ABGD with 72.84% accuracy, and 81 species by threshold-NJ with 72.84% accuracy. The COII-376 dataset with only one sequence from NCBI contained 51 morphological species, and 53 hypothetic species were gleaned using the GMYC method with an accuracy rate of 83.02%, 48 species using PTP with 83.33% accuracy, and 50 species using ABGD with 82.00% accuracy. The Cytb-413 data contained 48 morphological species and generated 54 clusters by the GMYC method with an accuracy rate of 79.63%, 49 species using PTP with 81.63% accuracy, and 48 species using ABGD with 79.17% accuracy. There were 49 morphological species within gnd-396, and the putative species found using the GMYC approach was 46 with an accuracy rate of 86.96%, using the PTP method was 45 species with an accuracy rate of 84.44%, and using the ABGD analysis was 48 species with 87.50% accuracy. An analysis of COI-338, COII-338, Cytb-338, and gnd-338 were performed to compare the results of different genes with diverse methods under the same sample composition. There were 45 morphological species within the given 338 samples. The analysis results of various genes and accuracy rate were: COI-338 (GMYC: 47, 89.36%; PTP: 49, 85.71%; ABGD: 46, 91.30%), COII-338 (GMYC: 45, 93.33%; PTP: 45, 86.67%; ABGD: 45, 86.67%), Cytb-338 (GMYC: 50, 80.00%; PTP: 46, 80.43%; ABGD: 42, 83.33%), and gnd-338 (GMYC: 42, 92.86%; PTP: 41, 90.24%; ABGD: 44, 93.18%). The final results were all displayed in NJ trees (see Suppl. material 4–11) with bootstrap values. The tree topology should be regarded only as distance affinity and not a phylogenetic relationship.

## Discussion

### The appropriate DNA barcoding and suitable analysis method to Chaitophorinae

COI has not been the only gene marker used for aphid DNA barcoding, other genes from the mitochondrial genome and from endosymbionts having been used for various aphid groups (Chen et al. 2012, Chen et al. 2013a, Liu et al. 2013, Tang et al. 2015) . Additionally, many analytical methods have been used in DNA barcoding. Here, we focused on COI, COII, Cytb, and gnd genes, and applied four methods, threshold with NJ, ABGD, GMYC, and PTP, to delimitate species of Chaitophorinae.

For different genes, the amplification efficiency was COI (100%) > Cytb (97%) > gnd (93%) > COII (88%). Within the 338-sample dataset, the difference in values between the smallest interspecific distance and average coalescent depth were unequal in different groups. For *Chaitophorus*, *Periphyllus*, and *Chaetosiphella* (Table 1), the difference values were COI > Cytb > COII > gnd, gnd > COI > COII > Cytb, and gnd > COI > COII = Cytb, respectively. From the overlap region and total frequency (Figure 1), COI and COII were similar with a narrower overlap region and less frequency than Cytb. Although the overlap span of gnd was sufficient, its total frequency in that region was slightly larger. Therefore, the COI and COII genes may be better markers for DNA barcoding.

The most important factor in choosing the delimitation method was the identification accuracy within different genes. Therefore, a better approach means higher identification accuracy and a greater range of application with various genes. The accuracy of GMYC, PTP, and ABGD within COI-338, COII-338, Cytb-338, and gnd-338 were ABGD (91.30%) > GMYC (89.36%) > PTP (85.71%), GMYC (93.33%) > PTP = ABGD (86.67%), ABGD (83.33%) > PTP (80.43%) > GMYC (80.00%), and ABGD (93.18%) > GMYC (92.86%) > PTP (90.24%), respectively (Table 2). In Chaitophorinae, the ABGD was a much better analytical method, and GMYC was also better than PTP in tree-based approaches. Considering that the analysis of ABGD required prior intraspecific distance, a tree-based method needed to be employed concurrently. A tree-based approach should be crosschecked against a non-tree-based approach within species delimitation studies (Fontaneto et al. 2015). As different methods may yield inconformity conclusions (Carstens et al. 2013), the accurate identification of species requires further integrative analysis (Puillandre et al. 2012b, Pante et al. 2015). Herein, a brief investigation of the morphological characteristics combined with a distance-based method of ABGD and a tree-based method of GMYC may be a very suitable pattern for species delimitation and the rapid identification of Chaitophorinae.

### DNA barcoding may reveal population differentiation driven by geographical distribution

**Figure 2.**
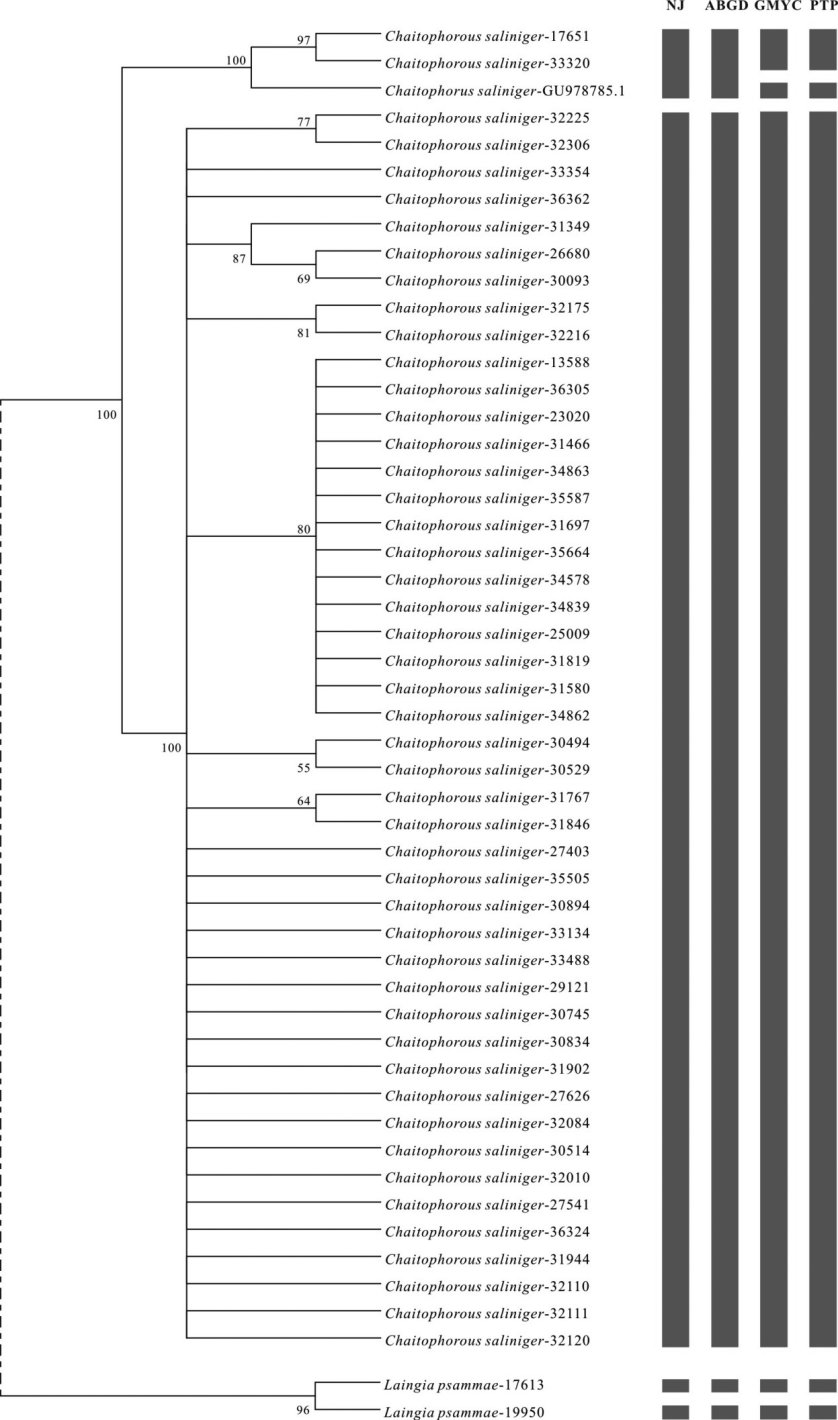
The analysis results of some species from the COI-670 dataset. The analysis results based on other genes were almost identical. The NJ tree was constructed based on the Kimura 2-parameter (K2P) model with a bootstrap value over 50% displayed. The gray blocks behind the tree represent the putative species, which means that the taxa in the tree corresponding to a single block are in one putative species. The number of blocks express the number of putative species using this method. **A** *Chaitophorus saliniger* **B** *Laingia psammae*.

*Chaitophorus saliniger* Shinji is an important pest on willows in East Asia. Based on the topology structure and results of analysis with different methods (Figure 2A, Suppl. material 4–11), all samples of this species were divided into two clades which could be regarded as two different species. However, based on the morphological characteristics, all samples should be *Chaitophorus saliniger*. On examining the geographical distribution information of all samples, we found that one of the clades consisted of two samples (Nos. 17651 and 33320) of *Chaitophorus saliniger* collected from Northeast China, Heilongjiang Province, and the voucher of another sequence (*Chaitophorus saliniger*-GU978785.1, a downloaded sequence from NCBI) from the Korean Peninsula. The location sites of these three samples were all at a relatively high latitude. All samples in the other clade were not from the aforementioned regions. So, within *Chaitophorus saliniger*, a population differentiation emerged among the samples from different locations. The genetic divergences between the two clades for COI, Cytb, and gnd were 0.043, 0.039, and 0.020, respectively, which could be regarded as interspecific distances. Although the morphological characteristics of all samples were similar, differentiation at the gene level seems to have occurred between northern and southern populations. Similar genetic differentiation between these populations has also been demonstrated for a nuclear gene, EF-1α (Fang et al. 2016).

In a similar manner, two samples (Nos. 17613 and 19950) of *Laingia psammae* Theobald were divided into two independent clades (Figure 2B), a result supported at all genes and using different approaches. Sample No. 17613 was from Jilin, northeastern China, whereas sample No. 19950 was from Xinjiang, northwestern China. The genetic distances of three genes (COI, COII and Cytb) between the two samples were 0.064, 0.068, and 0.062, respectively, which reach the level of species (Wang and Qiao 2009). Therefore, differentiation between northeastern and northwestern populations in *Laingia psammae* exists.

From the topology structures and the constructed consequences of threshold with NJ, ABGD, GMYC, and PTP, we observed that population differentiation was clearly present within both *Chaitophorus saliniger* and *Laingia psammae*. Similar findings have been reported in other aphid species (Lee et al. 2011, Wang et al. 2011). The prominent differences among populations may even be an indication of cryptic species (Bickford et al. 2007). Speciation is a long and continuous process, and cryptic species are not easily explained. Within the process, the incipient species may hold for millions of years (Avise and Walker 1998). Therefore, cryptic species need further study with more samples, combined with morphological characteristics and biological information.

## Conclusions

In this work, the DNA barcoding of Chaitophorinae aphids was investigated. Three mitochondrial genes and one endosymbiont gene were used to calculate and compare the genetic distances within different datasets. For the delimitation of species, two distance-based methods, threshold with NJ and ABGD, and two tree-based approaches, GMYC and PTP were employed. The interspecific genetic divergence was clearly greater than intraspecific divergence in the four molecular markers. Additionally, the COI and COII genes were more suitable as Chaitophorinae DNA barcoding markers. Based on the data for *Chaitophorus saliniger* and *Laingia psammae*, DNA barcoding may reveal population differentiation driven by geographical distribution.

## Supplementary materials

Supplementary material 1 Table S1 Data type: specimens data Explanation note: Sample information. This dataset is made available under the Open Database License (http://opendatacommons.org/licenses/odbl/1.0/). The Open Database License (ODbL) is a license agreement intended to allow users to freely share, modify, and use this Dataset while maintaining this same freedom for others, provided that the original source and author(s) are credited.Xi-Chao Zhu, Jing Chen, Rui Chen, Li-Yun Jiang, Ge-Xia Qiao

Supplementary material 2 Table S2 Data type: molecular data Explanation note: Primer information. This dataset is made available under the Open Database License (http://opendatacommons.org/licenses/odbl/1.0/). The Open Database License (ODbL) is a license agreement intended to allow users to freely share, modify, and use this Dataset while maintaining this same freedom for others, provided that the original source and author(s) are credited.Xi-Chao Zhu, Jing Chen, Rui Chen, Li-Yun Jiang, Ge-Xia Qiao

Supplementary material 3 Table S3 Data type: molecular data Explanation note: The analysis results with ABGD of all datasets. This dataset is made available under the Open Database License (http://opendatacommons.org/licenses/odbl/1.0/). The Open Database License (ODbL) is a license agreement intended to allow users to freely share, modify, and use this Dataset while maintaining this same freedom for others, provided that the original source and author(s) are credited.Xi-Chao Zhu, Jing Chen, Rui Chen, Li-Yun Jiang, Ge-Xia Qiao

Supplementary material 4 Figure S1 Data type: molecular data Explanation note: The analysis results of dataset COI-670. This dataset is made available under the Open Database License (http://opendatacommons.org/licenses/odbl/1.0/). The Open Database License (ODbL) is a license agreement intended to allow users to freely share, modify, and use this Dataset while maintaining this same freedom for others, provided that the original source and author(s) are credited.Xi-Chao Zhu, Jing Chen, Rui Chen, Li-Yun Jiang, Ge-Xia Qiao

Supplementary material 5 Figure S2 Data type: molecular data Explanation note: The analysis results of dataset COII-376. This dataset is made available under the Open Database License (http://opendatacommons.org/licenses/odbl/1.0/). The Open Database License (ODbL) is a license agreement intended to allow users to freely share, modify, and use this Dataset while maintaining this same freedom for others, provided that the original source and author(s) are credited.Xi-Chao Zhu, Jing Chen, Rui Chen, Li-Yun Jiang, Ge-Xia Qiao

Supplementary material 6 Figure S3 Data type: molecular data Explanation note: The analysis results of dataset Cytb-413. This dataset is made available under the Open Database License (http://opendatacommons.org/licenses/odbl/1.0/). The Open Database License (ODbL) is a license agreement intended to allow users to freely share, modify, and use this Dataset while maintaining this same freedom for others, provided that the original source and author(s) are credited.Xi-Chao Zhu, Jing Chen, Rui Chen, Li-Yun Jiang, Ge-Xia Qiao

Supplementary material 7 Figure S4 Data type: molecular data Explanation note: The analysis results of dataset gnd-396. This dataset is made available under the Open Database License (http://opendatacommons.org/licenses/odbl/1.0/). The Open Database License (ODbL) is a license agreement intended to allow users to freely share, modify, and use this Dataset while maintaining this same freedom for others, provided that the original source and author(s) are credited.Xi-Chao Zhu, Jing Chen, Rui Chen, Li-Yun Jiang, Ge-Xia Qiao

Supplementary material 8 Figure S5 Data type: molecular data Explanation note: The analysis results of dataset COI-338. This dataset is made available under the Open Database License (http://opendatacommons.org/licenses/odbl/1.0/). The Open Database License (ODbL) is a license agreement intended to allow users to freely share, modify, and use this Dataset while maintaining this same freedom for others, provided that the original source and author(s) are credited.Xi-Chao Zhu, Jing Chen, Rui Chen, Li-Yun Jiang, Ge-Xia Qiao

Supplementary material 9 Figure S6 Data type: molecular data Explanation note: The analysis results of dataset COII-338. This dataset is made available under the Open Database License (http://opendatacommons.org/licenses/odbl/1.0/). The Open Database License (ODbL) is a license agreement intended to allow users to freely share, modify, and use this Dataset while maintaining this same freedom for others, provided that the original source and author(s) are credited.Xi-Chao Zhu, Jing Chen, Rui Chen, Li-Yun Jiang, Ge-Xia Qiao

Supplementary material 10 Figure S7 Data type: molecular data Explanation note: The analysis results of dataset Cytb-338. This dataset is made available under the Open Database License (http://opendatacommons.org/licenses/odbl/1.0/). The Open Database License (ODbL) is a license agreement intended to allow users to freely share, modify, and use this Dataset while maintaining this same freedom for others, provided that the original source and author(s) are credited.Xi-Chao Zhu, Jing Chen, Rui Chen, Li-Yun Jiang, Ge-Xia Qiao

Supplementary material 11 Figure S8 Data type: molecular data Explanation note: The analysis results of dataset gnd-338. This dataset is made available under the Open Database License (http://opendatacommons.org/licenses/odbl/1.0/). The Open Database License (ODbL) is a license agreement intended to allow users to freely share, modify, and use this Dataset while maintaining this same freedom for others, provided that the original source and author(s) are credited.Xi-Chao Zhu, Jing Chen, Rui Chen, Li-Yun Jiang, Ge-Xia Qiao
