# Supplementary material for: DNA barcoding and species delimitation of Chaitophorinae (Hemiptera, Aphididae)
Source: Zookeys. 2017 Feb 14;(656):25–50. doi: 10.3897/zookeys.656.11440 (PMC5345361; doi:10.3897/zookeys.656.11440)
Supplement: Supplementary material 1 — Table S1 [file zookeys-656-025-s001.docx]

**Table S1. Samples information**

| **voucher** | **species** | **host plant** | **COI/GenBank accession no.** | **COII/GenBank accession no.** | **cytb/GenBank accession no.** | **gnd/GenBank accession no.** | **collection site** |
| --- | --- | --- | --- | --- | --- | --- | --- |
| 13347 | *Chaitophorus populialbae* | *Populus* sp. | KX679969 | KX680327 | KX680702 | KX681115 | China: Beijing |
| 13349 | *Chaitophorus populeti* | *Populus* sp. | KX679970 | KX680328 | KX680703 | KX681116 | China: Beijing |
| 13458 | *Periphyllus diacerivorus* | *Acer* sp. | KX679971 | KX680329 | KX680704 | KX681117 | China: Beijing |
| 13459 | *Periphyllus diacerivorus* | *Acer* sp. | KX679972 | KX680330 | KX680705 | KX681118 | China: Beijing |
| 13477 | *Periphyllus koelreuteriae* | *Koelreuteria paniculata* | KX679973 | KX680331 | KX680706 | KX681119 | China: Beijing |
| 13505 | *Chaitophorus indicus* | *Populus* sp. | KX620551** | KX680332 | KX680707 | KX620698** | China: Xizang |
| 13588 | *Chaitophorus saliniger* | *Salix babylonica* | KT237550* | - | KX680708 | KT236963** | China: Qinghai |
| 13647 | *Chaitophorus* sp.4 | *Populus* sp. | KX620559** | KX680333 | KX680709 | KX620706** | China: Xinjiang |
| 13661 | *Lambersaphis pruinosa* | *Populus nigra* | KX679974 | KX680334 | KX680710 | KX681120 | China: Xinjiang |
| 13662 | *Chaitophorus populeti* | *Populus nigra* | KX679975 | KX680335 | KX680711 | KX681121 | China: Xinjiang |
| 13663 | *Lambersaphis pruinosa* | *Salix* sp. | KX679976 | KX680336 | KX680712 | KX681122 | China: Xinjiang |
| 13664 | *Chaitophorus populeti* | *Populus* sp. | KX679977 | KX680337 | KX680713 | KX681123 | China: Xinjiang |
| 13730 | *Chaitophorus pruinosae* | *Populus euphratica* | KX679978 | KX680338 | KX680714 | - | China: Xinjiang |
| 13748 | *Chaitophorus* sp.1 | *Populus* sp. | KX679979 | KX680339 | KX680715 | KX681124 | China: Xinjiang |
| 13783 | *Chaitophorus saliapterus quinquemaculatus* | *Salix* sp. | KX679980 | KX680340 | KX680716 | KX681125 | China: Xinjiang |
| 13793 | *Chaitophorus saliapterus quinquemaculatus* | *Populus euphratica* | KX620529** | KX680341 | KX680717 | KX620676** | China: Xinjiang |
| 13843 | *Chaitophorus salijaponicus szelegiewiczi* | *Salix* sp. | KX679981 | KX680342 | KX680718 | KX681126 | China: Xinjiang |
| 13861 | *Chaitophorus populialbae yomefuri* | *Populus* sp. | KX679982 | KX680343 | KX680719 | - | China: Xinjiang |
| 13862 | *Chaitophorus* sp.1 | *Populus* sp. | KX679983 | KX680344 | KX680720 | KX681127 | China: Xinjiang |
| 13881 | *Chaitophorus* sp.4 | *Armeniaca vulgaris* | KX679984 | KX680345 | KX680721 | KX681128 | China: Xinjiang |
| 13900 | *Chaitophorus populeti* | *Populus* sp. | KX679985 | KX680346 | KX680722 | KX681129 | China: Xinjiang |
| 13925 | *Chaitophorus populialbae yomefuri* | *Populus* sp. | KX679986 | KX680347 | KX680723 | KX681130 | China: Xinjiang |
| 13932 | *Chaitophorus populialbae yomefuri* | *Populus* sp. | KX679987 | KX680348 | KX680724 | KX681131 | China: Xinjiang |
| 13933 | *Chaitophorus* sp.1 | *Populus* sp. | KX679988 | KX680349 | KX680725 | KX681132 | China: Xinjiang |
| 13951 | *Chaitophorus populialbae yomefuri* | *Populus* sp. | KX679989 | KX680350 | KX680726 | KX681133 | China: Xinjiang |
| 13962 | *Chaitophorus populialbae yomefuri* | *Populus* sp. | KX679990 | KX680351 | KX680727 | KX681134 | China: Xinjiang |
| 14020 | *Chaitophorus* sp.1 | *Populus* sp. | KX679991 | KX680352 | KX680728 | KX681135 | China: Xinjiang |
| 14047 | *Chaitophorus* sp.9 | *Populus* sp. | KX679992 | KX680353 | KX680729 | KX681136 | China: Qinghai |
| 14048 | *Chaitophorus* sp.9 | *Populus* sp. | KX679993 | KX680354 | KX680730 | KX681137 | China: Qinghai |
| 14070 | *Chaitophorus* sp.9 | *Populus* sp. | KX620558** | KX680355 | KX680731 | KX620705** | China: Qinghai |
| 14071 | *Chaitophorus* sp.9 | *Populus* sp. | KX679994 | KX680356 | KX680732 | KX681138 | China: Qinghai |
| 14072 | *Chaitophorus* sp.9 | *Populus* sp. | KX679995 | KX680357 | KX680733 | KX681139 | China: Qinghai |
| 14107 | *Chaitophorus* sp.9 | *Populus* sp. | KX679996 | KX680358 | KX680734 | KX681140 | China: Qinghai |
| 14108 | *Chaitophorus* sp.9 | *Populus* sp. | KX679997 | KX680359 | KX680735 | KX681141 | China: Qinghai |
| 14211 | *Periphyllus diacerivorus* | *Acer mono* | KX679998 | KX680360 | KX680736 | KX681142 | China: Beijing |
| 14212 | *Chaitophorus populialbae* | *Populus* sp. | KX679999 | KX680361 | KX680737 | KX681143 | China: Beijing |
| 14213 | *Periphyllus acerihabitans* | *Acer mono* | KX680000 | KX680362 | KX680738 | KX681144 | China: Beijing |
| 14214 | *Chaitophorus populialbae* | *Populus tomentosa* | KX680001 | KX680363 | KX680739 | KX681145 | China: Beijing |
| 14220 | *Periphyllus koelreuteriae* | *Koelreuteria paniculata* | KX680002 | KX680364 | KX680740 | KX681146 | China: Beijing |
| 14258 | *Chaitophorus populeti* | *Populus* sp. | KX680003 | KX680365 | KX680741 | KX681147 | China: Beijing |
| 14280 | *Chaitophorus* sp.2 | *Populus* sp. | KX620533** | KX680366 | KX680742 | KX620680** | China: Hebei |
| 14343 | *Chaitophorus populeti* | *Populus* sp. | KX680004 | KX680367 | KX680743 | - | China: Beijing |
| 14371 | *Chaitophorus populialbae* | *Populus tomentosa* | KX680005 | KX680368 | KX680744 | KX681148 | China: Beijing |
| 14726 | *Periphyllus* sp.6 | *Acer* sp. | KX680006 | KX680369 | KX680745 | KX681149 | China: Fujian |
| 14753 | *Periphyllus bengalensis* | *Acer* sp. | KX680007 | KX680370 | KX680746 | KX681150 | China: Fujian |
| 14764 | *Trichaitophorus recurvispinus* | *Acer oliverianum* | KX620564** | KX680371 | KX680747 | KX681151 | China: Fujian |
| 15127 | *Chaitophorus populeti* | *Populus* sp. | KX680008 | KX680372 | KX680748 | KX681152 | China: Beijing |
| 15157 | *Periphyllus diacerivorus* | *Acer mono* | KX680009 | KX680373 | KX680749 | KX681153 | China: Beijing |
| 15653 | *Chaitophorus populeti* | *Populus* sp. | KX620534** | KX680374 | KX680750 | KX620681** | China: Guizhou |
| 16067 | *Periphyllus acerihabitans* | *Acer* sp. | KX680010 | KX680375 | KX680751 | KX681154 | China: Beijing |
| 16068 | *Periphyllus diacerivorus* | *Acer* sp. | KX680011 | KX680376 | KX680752 | KX681155 | China: Beijing |
| 16085 | *Periphyllus acerihabitans* | *Acer truncatum* | KX680012 | KX680377 | KX680753 | KX681156 | China: Beijing |
| 16091 | *Periphyllus kuwanaii* | *Acer truncatum* | KX680013 | KX680378 | KX680754 | KX681157 | China: Beijing |
| 16099 | *Chaitophorus leucomelas* | *Populus* sp. | KX680014 | KX680379 | KX680755 | KX681158 | China: Beijing |
| 16142 | *Chaitophorus populeti* | *Populus tomentosa* | KX680015 | KX680380 | KX680756 | KX681159 | China: Beijing |
| 16158 | *Periphyllus diacerivorus* | *Acer mono* | KX680016 | KX680381 | KX680757 | KX681160 | China: Beijing |
| 16165 | *Chaitophorus populeti* | *Populus* sp. | KX680017 | KX680382 | KX680758 | KX681161 | China: Beijing |
| 16573 | *Chaitophorus populeti* | *Populus tomentosa* | KX680018 | KX680383 | KX680759 | KX681162 | China: Beijing |
| 16587 | *Chaitophorus populialbae* | *Populus* sp. | KX620539** | KX680384 | KX680760 | KX620686** | China: Xinjiang |
| 16635 | *Chaitophorus leucomelas* | *Populus canadensis* | KX680019 | KX680385 | KX680761 | KX681163 | China: Xinjiang |
| 16657 | *Chaitophorus leucomelas* | *Populus canadensis* | KX680020 | KX680386 | KX680762 | KX681164 | China: Xinjiang |
| 16663 | *Chaitophorus leucomelas* | *Populus canadensis* | KX680021 | KX680387 | KX680763 | KX681165 | China: Xinjiang |
| 16668 | *Chaitophorus leucomelas* | *Populus canadensis* | KX680022 | KX680388 | KX680764 | KX681166 | China: Xinjiang |
| 16697 | *Chaitophorus populeti* | *Populus tomentosa* | KX680023 | KX680389 | KX680765 | - | China: Xinjiang |
| 16715 | *Chaitophorus populeti* | *Populus tomentosa* | KX680024 | KX680390 | KX680766 | KX681167 | China: Xinjiang |
| 16720 | *Chaitophorus populeti* | *Populus tomentosa* | KX680025 | KX680391 | KX680767 | - | China: Xinjiang |
| 16731 | *Chaitophorus* sp.1 | *Populus tomentosa* | KX680026 | KX680392 | KX680768 | KX681168 | China: Xinjiang |
| 16742 | *Chaitophorus populeti* | *Populus canadensis* | KX680027 | KX680393 | KX680769 | - | China: Xinjiang |
| 16745 | *Chaitophorus populeti* | *Populus tomentosa* | KX680028 | KX680394 | KX680770 | - | China: Xinjiang |
| 16753 | *Chaitophorus* sp.1 | *Populus tomentosa* | KX680029 | KX680395 | KX680771 | KX681169 | China: Xinjiang |
| 16786 | *Chaitophorus* sp.1 | *Populus tomentosa* | KX680030 | KX680396 | KX680772 | KX681170 | China: Xinjiang |
| 16800 | *Chaitophorus* sp.1 | *Populus tomentosa* | KX680031 | KX680397 | KX680773 | KX681171 | China: Xinjiang |
| 16840 | *Chaitophorus populeti* | *Populus tomentosa* | KX680032 | KX680398 | KX680774 | KX681172 | China: Xinjiang |
| 16843 | *Chaitophorus populeti* | *Populus tomentosa* | KX680033 | KX680399 | KX680775 | - | China: Xinjiang |
| 16867 | *Chaitophorus* sp.1 | *Populus tomentosa* | KX680034 | KX680400 | KX680776 | KX681173 | China: Xinjiang |
| 16868 | *Chaitophorus* sp.1 | *Populus tomentosa* | KX680035 | KX680401 | KX680777 | KX681174 | China: Xinjiang |
| 16876 | *Chaitophorus* sp.1 | *Populus tomentosa* | KX680036 | KX680402 | KX680778 | KX681175 | China: Xinjiang |
| 16880 | *Chaitophorus* sp.1 | *Populus tomentosa* | KX680037 | KX680403 | KX680779 | KX681176 | China: Xinjiang |
| 16895 | *Chaitophorus* sp.1 | *Populus tomentosa* | KX680038 | KX680404 | KX680780 | KX681177 | China: Xinjiang |
| 16907 | *Chaitophorus* sp.1 | *Populus tomentosa* | KX680039 | KX680405 | KX680781 | KX681178 | China: Xinjiang |
| 16913 | *Chaitophorus* sp.1 | *Populus* sp. | KX680040 | KX680406 | KX680782 | KX681179 | China: Xinjiang |
| 16917 | *Chaitophorus* sp.1 | *Populus tomentosa* | KX680041 | KX680407 | KX680783 | KX681180 | China: Xinjiang |
| 16948 | *Chaitophorus populeti* | *Populus* sp. | KX680042 | KX680408 | KX680784 | KX681181 | China: Shaanxi |
| 17454 | *Chaitophorus populeti* | *Populus* sp. | KX680043 | KX680409 | KX680785 | - | China: Hebei |
| 17470 | *Sipha* (*Rungsia*) *burakowskii* | Gramineae | KX680044 | KX680410 | KX680786 | KX681182 | China: Hebei |
| 17529 | *Chaitophorus populeti* | *Populus* sp. | KX680045 | KX680411 | KX680787 | - | China: Liaoning |
| 17556 | *Chaitophorus* sp.5 | *Salix* sp. | KX620560** | KX680412 | KX680788 | KX620707** | China: Liaoning |
| 17601 | *Chaitophorus populeti* | *Populus* sp. | KX680046 | KX680413 | KX680789 | - | China: Jilin |
| 17613 | *Laingia psammae* | Gramineae | KX680047 | KX680414 | KX680790 | - | China: Jilin |
| 17646 | *Chaitophorus populeti* | *Populus* sp. | KX680048 | KX680415 | KX680791 | KX681183 | China: Heilongjiang |
| 17649 | *Chaitophorus populeti* | *Populus* sp. | KX680049 | KX680416 | KX680792 | KX681184 | China: Heilongjiang |
| 17651 | *Chaitophorus saliniger* | *Salix* sp. | KT237614* | - | KX680793 | KT237027** | China: Heilongjiang |
| 17692 | *Chaitophorus populeti* | *Populus* sp. | KX680050 | KX680417 | KX680794 | KX681185 | China: Heilongjiang |
| 17709 | *Chaitophorus populeti* | *Populus* sp. | KX620535** | KX680418 | KX680795 | KX620682** | China: Heilongjiang |
| 17717 | *Chaitophorus populeti* | *Populus* sp. | KX680051 | KX680419 | KX680796 | - | China: Heilongjiang |
| 17956 | *Sipha* (*Rungsia*) sp. | Gramineae | KX680052 | KX680420 | KX680797 | - | China: Inner Mongolia |
| 18633 | *Periphyllus* sp.6 | *Acer cinnamomifolium* | KX680053 | KX680421 | KX680798 | KX681186 | China: Guangdong |
| 18822 | *Periphyllus kuwanaii* | *Acer truncatum* | KX680054 | KX680422 | KX680799 | KX681187 | China: Beijing |
| 18823 | *Periphyllus acerihabitans* | *Acer truncatum* | KX680055 | KX680423 | KX680800 | KX681188 | China: Beijing |
| 18842 | *Periphyllus diacerivorus* | *Acer truncatum* | KX680056 | KX680424 | KX680801 | KX681189 | China: Beijing |
| 19415 | *Chaitophorus populeti* | *Populus* sp. | KX680057 | KX680425 | KX680802 | KX681190 | China: Beijing |
| 19418 | *Chaitophorus populeti* | *Populus simonii* | KX680058 | KX680426 | KX680803 | KX681191 | China: Beijing |
| 19419 | *Chaitophorus tremulae* | *Populus simonii* | KX680059 | KX680427 | KX680804 | KX681192 | China: Beijing |
| 19464 | *Chaitophorus populeti* | *Populus* sp. | KX680060 | KX680428 | KX680805 | KX681193 | China: Beijing |
| 19472 | *Periphyllus kuwanaii* | *Acer mono* | KX680061 | KX680429 | KX680806 | KX681194 | China: Beijing |
| 19803 | *Chaitophorus* sp.1 | *Populus* sp. | KX680062 | KX680430 | KX680807 | KX681195 | China: Xinjiang |
| 19806 | *Chaitophorus* sp.1 | *Populus* sp. | KX680063 | KX680431 | KX680808 | KX681196 | China: Xinjiang |
| 19817 | *Chaitophorus pruinosae* | *Populus euphratica* | KX680064 | KX680432 | KX680809 | KX681197 | China: Xinjiang |
| 19821 | *Chaitophorus pruinosae* | *Salix* sp. | KX680065 | KX680433 | KX680810 | KX681198 | China: Xinjiang |
| 19838 | *Chaitophorus leucomelas* | *Populus* sp. | KX680066 | KX680434 | KX680811 | KX681199 | China: Xinjiang |
| 19842 | *Chaitophorus leucomelas* | *Populus* sp. | KX680067 | KX680435 | KX680812 | KX681200 | China: Xinjiang |
| 19864 | *Chaitophorus pruinosae* | *Populus euphratica* | KX680068 | KX680436 | KX680813 | KX681201 | China: Xinjiang |
| 19915 | *Chaitophorus leucomelas* | *Populus* sp. | KX680069 | KX680437 | KX680814 | KX681202 | China: Xinjiang |
| 19950 | *Laingia psammae* | Gramineae | KX680070 | KX680438 | KX680815 | - | China: Xinjiang |
| 20003 | *Chaitophorus leucomelas* | *Populus* sp. | KX620524** | KX680439 | - | KX620671** | China: Xinjiang |
| 20070 | *Chaitophorus pruinosae* | *Populus euphratica* | KX620548** | KX680440 | KX680816 | KX620695** | China: Xinjiang |
| 20575 | *Chaitophorus populeti* | *Populus* sp. | KX680071 | KX680441 | KX680817 | KX681203 | China: Xinjiang |
| 20697 | *Chaitophorus populeti* | *Populus* sp. | KX680072 | KX680442 | KX680818 | - | China: Xinjiang |
| 20710 | *Chaitophorus populeti* | *Populus* sp. | KX680073 | KX680443 | KX680819 | KX681204 | China: Xinjiang |
| 20714 | *Chaitophorus* sp.9 | *Populus* sp. | KX680074 | KX680444 | KX680820 | KX681205 | China: Gansu |
| 21181 | *Chaitophorus populeti* | *Populus* sp. | KX680075 | KX680445 | KX680821 | KX681206 | China: Ningxia |
| 21264 | *Chaitophorus nigricantis* | *Salix* sp. | KX680076 | KX680446 | KX680822 | KX681207 | China: Jilin |
| 21279 | *Chaitophorus capreae* | *Salix* sp. | KX620547** | KX680447 | KX680823 | KX620694** | China: Jilin |
| 21282 | *Chaitophorus nigricantis* | *Populus* sp. | KX680077 | KX680448 | KX680824 | KX681208 | China: Jilin |
| 21332 | *Chaitophorus truncatus* | *Salix* sp. | KX620552** | KX680449 | KX680825 | KX620699** | Korea |
| 21676 | *Chaitophorus populeti* | *Populus* sp. | KX680078 | KX680450 | KX680826 | KX681209 | China: Beijing |
| 22197 | *Chaitophorus* sp.8 | *Populus* sp. | KX680079 | KX680451 | KX680827 | KX681210 | China: Beijing |
| 22198 | *Periphyllus koelreuteriae* | *Koelreuteria paniculata* | KX680080 | KX680452 | KX680828 | KX681211 | China: Beijing |
| 22201 | *Chaitophorus* sp.8 | *Populus* sp. | KX680081 | KX680453 | KX680829 | KX681212 | China: Beijing |
| 22205 | *Chaitophorus populialbae* | *Populus* sp. | KX680082 | KX680454 | KX680830 | KX681213 | China: Beijing |
| 22328 | *Chaitophorus* sp.9 | *Populus simonii* | KX620554** | KX680455 | KX680831 | KX620701** | China: Jilin |
| 22367 | *Periphyllus bulgaricus* | *Acer* sp. | KX680083 | KX680456 | KX680832 | KX681214 | China: Jilin |
| 22626 | *Periphyllus* sp.5 | *Acer* sp. | KX680084 | KX680457 | KX680833 | KX681215 | China: Sichuan |
| 22659 | *Periphyllus* sp.5 | *Acer* sp. | KX680085 | KX680458 | KX680834 | KX681216 | China: Sichuan |
| 22735 | *Chaitophorus* sp.8 | *Populus* sp. | KX680086 | KX680459 | KX680835 | KX681217 | Mongolia |
| 22753 | *Chaitophorus populeti* | *Populus* sp. | KX680087 | KX680460 | KX680836 | KX681218 | Mongolia |
| 22773 | *Chaitophorus populeti* | *Populus* sp. | KX680088 | KX680461 | KX680837 | KX681219 | Mongolia |
| 22822 | *Chaitophorus leucomelas* | *Populus* sp. | KX680089 | KX680462 | KX680838 | KX681220 | China: Beijing |
| 22852 | *Chaitophorus populeti* | *Populus* sp. | KX680090 | KX680463 | KX680839 | - | China: Beijing |
| 22870 | *Chaitophorus* sp.8 | *Populus* sp. | KX680091 | KX680464 | KX680840 | KX681221 | China: Beijing |
| 22877 | *Chaitophorus populeti* | *Populus* sp. | KX680092 | KX680465 | KX680841 | KX681222 | China: Beijing |
| 22878 | *Chaitophorus populeti* | *Populus* sp. | KX680093 | KX680466 | KX680842 | KX681223 | China: Beijing |
| 23018 | *Chaitophorus populialbae* | *Populus* sp. | KX680094 | KX680467 | KX680843 | KX681224 | China: Gansu |
| 23020 | *Chaitophorus saliniger* | *Salix* sp. | KX680095 | - | KX680844 | KX681225 | China: Gansu |
| 23237 | *Periphyllus* sp.3 | *Acer* sp. | KX680096 | KX680468 | KX680845 | KX681226 | China: Heilongjiang |
| 23416 | *Chaitophorus populeti* | *Populus* sp. | KX680097 | KX680469 | KX680846 | KX681227 | China: Beijing |
| 23660 | *Chaitophorus* sp.1 | *Populus* sp. | KX680098 | KX680470 | KX680847 | KX681228 | China: Xinjiang |
| 23661 | *Chaitophorus populeti* | *Populus* sp. | KX620536** | KX680471 | KX680848 | KX620683** | China: Xinjiang |
| 23674 | *Chaitophorus populialbae* | *Populus* sp. | KX680099 | KX680472 | KX680849 | KX681229 | China: Beijing |
| 23684 | *Chaitophorus* sp.8 | *Populus* sp. | KX680100 | KX680473 | KX680850 | KX681230 | China: Beijing |
| 24668 | *Chaitophorus populeti* | *Populus* sp. | KX680101 | KX680474 | KX680851 | - | Mongolia |
| 24714 | *Chaitophorus populeti* | *Populus suaveolens* | KX680102 | KX680475 | KX680852 | - | Mongolia |
| 24746 | *Chaetosiphella stipae* | Gramineae | KX680103 | KX680476 | KX680853 | KX681231 | Mongolia |
| 24757 | *Chaitophorus saliapterus quinquemaculatus* | *Salix* sp. | KX680104 | KX680477 | KX680854 | KX681232 | Mongolia |
| 24796 | *Chaitophorus truncatus* | *Salix* sp. | KX620553** | KX680478 | KX680855 | KX620700** | Mongolia |
| 24797 | *Chaetosiphella stipae* | *Achnatherum splendens* | KX680105 | KX680479 | KX680856 | KX681233 | Mongolia |
| 24808 | *Chaetosiphella stipae* | *Stipa grandis* | KX680106 | KX680480 | KX680857 | KX681234 | Mongolia |
| 25009 | *Chaitophorus saliniger* | *Salix* sp. | KT237841* | - | KX680858 | KX620690** | China: Shaanxi |
| 25075 | *Chaitophorus populeti* | *Populus davidiana* | KX680107 | KX680481 | KX680859 | KX681235 | China: Inner Mongolia |
| 25076 | *Chaitophorus populeti* | *Populus davidiana* | KX680108 | KX680482 | KX680860 | KX681236 | China: Inner Mongolia |
| 25091 | *Chaetosiphella stipae* | *Stipa* sp. | KX680109 | KX680483 | KX680861 | KX681237 | China: Inner Mongolia |
| 25113 | *Chaitophorus populeti* | *Populus* sp. | KX680110 | KX680484 | KX680862 | - | China: Inner Mongolia |
| 25132 | *Chaitophorus populeti* | *Populus davidiana* | KX680111 | KX680485 | KX680863 | KX681238 | China: Inner Mongolia |
| 25138 | *Chaetosiphella longirostris* | *Stipa* sp. | KX680112 | KX680486 | KX680864 | KX681239 | China: Inner Mongolia |
| 25161 | *Chaetosiphella longirostris* | *Stipa* sp. | KX680113 | KX680487 | KX680865 | KX681240 | China: Inner Mongolia |
| 25163 | *Chaetosiphella stipae* | *Stipa* sp. | KX680114 | KX680488 | KX680866 | KX681241 | China: Inner Mongolia |
| 25174 | *Chaitophorus populeti* | *Populus* sp. | KX680115 | KX680489 | KX680867 | KX681242 | China: Inner Mongolia |
| 25179 | *Chaitophorus populeti* | *Populus* sp. | KX680116 | KX680490 | KX680868 | KX681243 | China: Inner Mongolia |
| 25182 | *Chaetosiphella stipae* | *Stipa* sp. | KX680117 | KX680491 | KX680869 | KX681244 | China: Inner Mongolia |
| 25191 | *Chaetosiphella stipae* | *Stipa* sp. | KX680118 | KX680492 | KX680870 | KX681245 | China: Inner Mongolia |
| 25192 | *Chaitophorus populeti* | *Populus* sp. | KX680119 | KX680493 | KX680871 | KX681246 | China: Inner Mongolia |
| 25216 | *Chaitophorus pruinosae* | *Salix* sp. | KX680120 | KX680494 | KX680872 | KX681247 | China: Inner Mongolia |
| 25279 | *Chaitophorus populeti* | *Populus cathayana* | KX680121 | KX680495 | KX680873 | - | China: Inner Mongolia |
| 25358 | *Chaetosiphella stipae* | Gramineae | KX680122 | KX680496 | KX680874 | KX681248 | China: Inner Mongolia |
| 25370 | *Chaetosiphella stipae* | Gramineae | KX680123 | KX680497 | KX680875 | KX681249 | China: Inner Mongolia |
| 25397 | *Chaitophorus populeti* | *Populus cathayana* | KX680124 | KX680498 | KX680876 | KX681250 | China: Inner Mongolia |
| 25409 | *Chaitophorus populeti* | *Populus cathayana* | KX680125 | KX680499 | KX680877 | KX681251 | China: Inner Mongolia |
| 25447 | *Chaitophorus populeti* | *Populus* sp. | KX680126 | KX680500 | KX680878 | KX681252 | China: Beijing |
| 25449 | *Chaitophorus populeti* | *Populus* sp. | KX680127 | KX680501 | KX680879 | KX681253 | China: Beijing |
| 25511 | *Chaitophorus populeti* | *Populus* sp. | KX680128 | KX680502 | KX680880 | KX681254 | China: Beijing |
| 25542 | *Chaitophorus populeti* | *Populus* sp. | KX680129 | KX680503 | KX680881 | KX681255 | China: Beijing |
| 25543 | *Chaitophorus populeti* | *Populus* sp. | KX680130 | KX680504 | KX680882 | KX681256 | China: Beijing |
| 25550 | *Chaitophorus populeti* | *Populus* sp. | KX680131 | KX680505 | KX680883 | KX681257 | China: Beijing |
| 26352 | *Periphyllus* sp.1 | *Acer buergerianum* | KX680132 | KX680506 | KX680884 | KX681258 | China: Jiangsu |
| 26370 | *Periphyllus* sp.4 | *Acer* sp. | KX680133 | - | KX680885 | KX681259 | China: Jiangsu |
| 26566 | *Periphyllus formosanus* | *Acer morrisonense* | KX680134 | KX680507 | KX680886 | KX681260 | China: Taiwan |
| 26569 | *Periphyllus formosanus* | *Acer morrisonense* | KX680135 | KX680508 | KX680887 | KX681261 | China: Taiwan |
| 26570 | *Periphyllus formosanus* | *Acer morrisonense* | KX680136 | KX680509 | KX680888 | KX681262 | China: Taiwan |
| 26680 | *Chaitophorus saliniger* | *Salix* sp. | KX620544** | - | KX680889 | KX620691** | China: Zhejiang |
| 27142 | *Chaitophorus leucomelas* | *Populus* sp. | KX620525** | KX680510 | KX680890 | KX620672** | Russia |
| 27296 | *Periphyllus diacerivorus* | *Acer* sp. | KX680137 | KX680511 | KX680891 | KX681263 | China: Liaoning |
| 27325 | *Chaitophorus populeti* | *Populus davidiana* | KX680138 | KX680512 | KX680892 | KX681264 | China: Inner Mongolia |
| 27326 | *Chaitophorus* sp.9 | *Populus davidiana* | KX620555** | KX680513 | KX680893 | KX620702** | China: Inner Mongolia |
| 27327 | *Chaitophorus populeti* | *Populus davidiana* | KX680139 | KX680514 | KX680894 | KX681265 | China: Inner Mongolia |
| 27403 | *Chaitophorus saliniger* | *Salix babylonica* | KX680140 | - | KX680895 | KX681266 | China: Beijing |
| 27405 | *Chaitophorus populeti* | *Populus* sp. | KX680141 | KX680515 | KX680896 | KX681267 | China: Beijing |
| 27407 | *Chaetosiphella stipae* | *Achnatherum splendens* | KX680142 | KX680516 | KX680897 | KX681268 | China: Inner Mongolia |
| 27410 | *Chaitophorus* sp.6 | *Populus* sp. | KX680143 | KX680517 | KX680898 | KX681269 | China: Inner Mongolia |
| 27417 | *Chaitophorus* sp.6 | *Populus* sp. | KX620540** | - | KX680899 | KX620687** | China: Inner Mongolia |
| 27496 | *Chaitophorus populeti* | *Populus* sp. | KX680144 | KX680518 | KX680900 | KX681270 | China: Beijing |
| 27534 | *Periphyllus kuwanaii* | *Acer* sp. | KX680145 | KX680519 | KX680901 | KX681271 | China: Beijing |
| 27541 | *Chaitophorus saliniger* | *Salix* sp. | KX620545** | - | KX680902 | KX620692** | China: Beijing |
| 27610 | *Chaitophorus populeti* | *Populus alba* | KX680146 | KX680520 | KX680903 | KX681272 | China: Inner Mongolia |
| 27626 | *Chaitophorus saliniger* | *Salix* sp. | KX680147 | - | KX680904 | KX681273 | China: Inner Mongolia |
| 27648 | *Chaitophorus populeti* | *Populus canadensis* | KX680148 | KX680521 | KX680905 | KX681274 | China: Inner Mongolia |
| 27659 | *Chaitophorus* sp.6 | *Populus alba* | KX680149 | KX680522 | KX680906 | KX681275 | China: Inner Mongolia |
| 27686 | *Chaetosiphella stipae* | Gramineae | KX680150 | KX680523 | KX680907 | KX681276 | China: Inner Mongolia |
| 27709 | *Chaitophorus populeti* | *Populus alba* | KX680151 | KX680524 | KX680908 | KX681277 | China: Inner Mongolia |
| 27744 | *Chaitophorus* sp.6 | *Populus alba* | KX680152 | KX680525 | KX680909 | KX681278 | China: Inner Mongolia |
| 27750 | *Chaetosiphella* sp. | *Stipa* sp. | KX680153 | KX680526 | KX680910 | KX681279 | China: Inner Mongolia |
| 27775 | *Chaitophorus populeti* | *Populus alba* | KX680154 | KX680527 | KX680911 | KX681280 | China: Inner Mongolia |
| 27972 | *Chaitophorus* sp.9 | *Populus* sp. | KX680155 | KX680528 | KX680912 | KX681281 | China: Yunnan |
| 28112 | *Chaitophorus populeti* | *Populus* sp. | KX620537** | KX680529 | KX680913 | KX620684** | China: Ningxia |
| 28246 | *Chaetosiphella stipae* | Gramineae | KX680156 | KX680530 | KX680914 | KX681282 | China: Inner Mongolia |
| 28281 | *Sipha* (*Rungsia*) *burakowskii* | *Leymus chinensis* | KX680157 | KX680531 | KX680915 | KX681283 | China: Inner Mongolia |
| 28282 | *Chaetosiphella stipae* | *Stipa* sp. | KX680158 | KX680532 | KX680916 | - | China: Inner Mongolia |
| 28283 | *Chaetosiphella stipae* | Gramineae | KX680159 | KX680533 | KX680917 | KX681284 | China: Inner Mongolia |
| 28296 | *Sipha* (*Rungsia*) *burakowskii* | Gramineae | KX680160 | KX680534 | KX680918 | KX681285 | China: Inner Mongolia |
| 28306 | *Chaitophorus populeti* | *Populus alba* | KX680161 | KX680535 | KX680919 | KX681286 | China: Inner Mongolia |
| 28314 | *Chaitophorus leucomelas* | *Populus* sp. | KX680162 | KX680536 | KX680920 | KX681287 | China: Inner Mongolia |
| 28341 | *Chaitophorus tremulae* | *Populus* sp. | KX680163 | KX680537 | KX680921 | KX681288 | China: Inner Mongolia |
| 28393 | *Periphyllus diacerivorus* | *Acer* sp. | KX680164 | KX680538 | KX680922 | KX681289 | China: Beijing |
| 28403 | *Chaitophorus* sp.5 | *Salix* sp. | KX620561** | KX680539 | KX680923 | KX620708** | China: Beijing |
| 28673 | *Chaitophorus populeti* | *Populus* sp. | KX680165 | KX680540 | KX680924 | KX681290 | China: Beijing |
| 28697 | *Chaetosiphella stipae* | Gramineae | KX680166 | KX680541 | KX680925 | KX681291 | China: Inner Mongolia |
| 28717 | *Chaitophorus leucomelas* | *Populus simonii* | KX620526** | KX680542 | KX680926 | KX620673** | China: Inner Mongolia |
| 28736 | *Chaitophorus leucomelas* | *Populus alba* | KX680167 | - | - | - | China: Inner Mongolia |
| 28740 | *Chaetosiphella stipae* | Gramineae | KX680168 | KX680543 | KX680927 | KX681292 | China: Inner Mongolia |
| 28761 | *Chaitophorus populeti* | *Populus alba* | KX680169 | KX680544 | KX680928 | KX681293 | China: Inner Mongolia |
| 28773 | *Chaitophorus pruinosae* | *Populus euphratica* | KX620549** | KX680545 | KX680929 | - | China: Inner Mongolia |
| 28778 | *Chaetosiphella stipae* | Gramineae | KX680170 | KX680546 | KX680930 | KX681294 | China: Inner Mongolia |
| 28788 | *Lambersaphis pruinosa* | *Populus euphratica* | KX680171 | KX680547 | KX680931 | KX681295 | China: Inner Mongolia |
| 28812 | *Chaetosiphella stipae* | *Stipa* sp. | KX680172 | KX680548 | KX680932 | KX681296 | China: Inner Mongolia |
| 28834 | *Periphyllus acerihabitans* | *Acer* sp. | KX680173 | KX680549 | KX680933 | KX681297 | China: Beijing |
| 28844 | *Chaitophorus populialbae* | *Populus simonii* | KX680174 | KX680550 | KX680934 | KX681298 | Mongolia |
| 28848 | *Chaitophorus populialbae* | *Populus simonii* | KX680175 | KX680551 | KX680935 | KX681299 | Mongolia |
| 28879 | *Chaetosiphella stipae* | *Achnatherum splendens* | KX680176 | KX680552 | KX680936 | KX681300 | Mongolia |
| 28908 | *Chaetosiphella stipae* | *Achnatherum splendens* | KX680177 | KX680553 | KX680937 | KX681301 | Mongolia |
| 29096 | *Chaitophorus populeti* | *Populus* sp. | KX680178 | KX680554 | KX680938 | KX681302 | China: Beijing |
| 29114 | *Chaitophorus populeti* | *Populus* sp. | KX680179 | KX680555 | KX680939 | KX681303 | China: Beijing |
| 29121 | *Chaitophorus saliniger* | *Salix* sp. | KX680180 | KX680556 | KX680940 | KX681304 | China: Beijing |
| 29155 | *Chaitophorus populeti* | *Populus* sp. | KX680181 | KX680557 | KX680941 | KX681305 | China: Beijing |
| 29165 | *Chaitophorus populeti* | *Populus* sp. | KX680182 | KX680558 | KX680942 | KX681306 | China: Beijing |
| 29171 | *Chaitophorus* sp.9 | *Populus* sp. | KX680183 | KX680559 | KX680943 | KX681307 | China: Qinghai |
| 29174 | *Chaitophorus* sp.9 | *Populus* sp. | KX680184 | KX680560 | KX680944 | KX681308 | China: Qinghai |
| 29180 | *Chaitophorus populeti* | *Populus alba* | KX680185 | KX680561 | KX680945 | KX681309 | China: Qinghai |
| 29204 | *Chaitophorus* sp.9 | *Populus simonii* | KX680186 | KX680562 | KX680946 | KX681310 | China: Qinghai |
| 29217 | *Chaitophorus* sp.9 | *Populus* sp. | KX680187 | KX680563 | KX680947 | KX681311 | China: Qinghai |
| 29249 | *Chaitophorus leucomelas* | *Populus* sp. | KX680188 | KX680564 | KX680948 | KX681312 | China: Inner Mongolia |
| 30093 | *Chaitophorus saliniger* | *Salix babylonica* | KT237858* | - | KX680949 | KT237271** | China: Sichuan |
| 30494 | *Chaitophorus saliniger* | *Salix* sp. | KT237859* | - | KX680950 | KT237272** | China: Yunnan |
| 30508 | *Chaitophorus populialbae* | *Populus* sp. | KX680189 | KX680565 | KX680951 | KX681313 | China: Shandong |
| 30514 | *Chaitophorus saliniger* | *Salix* sp. | KX680190 | - | KX680952 | KX681314 | China: Shandong |
| 30525 | *Chaitophorus populialbae* | *Populus* sp. | KX680191 | KX680566 | KX680953 | KX681315 | China: Hebei |
| 30529 | *Chaitophorus saliniger* | *Salix* sp. | KT237844* | - | KX680954 | KT237257** | China: Shaanxi |
| 30590 | *Periphyllus acerihabitans* | *Acer mono* | KX680192 | KX680567 | KX680955 | KX681316 | China: Beijing |
| 30692 | *Chaitophorus populeti* | *Populus* sp. | KX680193 | KX680568 | KX680956 | KX681317 | China: Shanxi |
| 30693 | *Chaitophorus populeti* | *Populus* sp. | KX680194 | KX680569 | KX680957 | KX681318 | China: Shanxi |
| 30714 | *Chaitophorus populialbae* | *Populus* sp. | KX680195 | KX680570 | KX680958 | KX681319 | China: Beijing |
| 30716 | *Chaitophorus populialbae* | *Populus* sp. | KX680196 | KX680571 | KX680959 | KX681320 | China: Beijing |
| 30717 | *Chaitophorus populeti* | *Populus* sp. | KX680197 | KX680572 | KX680960 | KX681321 | China: Beijing |
| 30719 | *Periphyllus koelreuteriae* | *Koelreuteria paniculata* | KX680198 | KX680573 | KX680961 | KX681322 | China: Beijing |
| 30721 | *Periphyllus koelreuteriae* | *Koelreuteria paniculata* | KX680199 | KX680574 | KX680962 | KX681323 | China: Beijing |
| 30729 | *Periphyllus acerihabitans* | *Acer* sp. | KX680200 | KX680575 | KX680963 | KX681324 | China: Beijing |
| 30735 | *Chaitophorus* sp.8 | *Populus* sp. | KX680201 | KX680576 | KX680964 | KX681325 | China: Beijing |
| 30745 | *Chaitophorus saliniger* | *Salix* sp. | KX680202 | - | KX680965 | KX681326 | China: Beijing |
| 30747 | *Chaitophorus populeti* | *Populus* sp. | KX680203 | KX680577 | KX680966 | KX681327 | China: Beijing |
| 30758 | *Periphyllus koelreuteriae* | *Koelreuteria paniculata* | KX680204 | KX680578 | KX680967 | KX681328 | China: Beijing |
| 30760 | *Chaitophorus populeti* | *Populus* sp. | KX680205 | KX680579 | KX680968 | KX681329 | China: Beijing |
| 30777 | *Chaitophorus populeti* | *Populus tomentosa* | KX680206 | KX680580 | KX680969 | KX681330 | China: Beijing |
| 30782 | *Chaitophorus populeti* | *Populus* sp. | KX680207 | KX680581 | KX680970 | KX681331 | China: Beijing |
| 30795 | *Chaitophorus populeti* | *Populus* sp. | KX680208 | KX680582 | KX680971 | KX681332 | China: Beijing |
| 30797 | *Chaitophorus leucomelas* | *Populus* sp. | KX680209 | KX680583 | KX680972 | KX681333 | China: Beijing |
| 30834 | *Chaitophorus saliniger* | *Salix* sp. | KT237536* | - | KX680973 | KT236949** | China: Beijing |
| 30839 | *Periphyllus koelreuteriae* | *Koelreuteria paniculata* | KX680210 | KX680584 | KX680974 | KX681334 | China: Beijing |
| 30841 | *Periphyllus koelreuteriae* | *Koelreuteria paniculata* | KX680211 | KX680585 | KX680975 | KX681335 | China: Beijing |
| 30857 | *Chaitophorus populeti* | *Populus* sp. | KX680212 | KX680586 | KX680976 | - | China: Hebei |
| 30858 | *Chaitophorus* sp.6 | *Populus* sp. | KX620541** | KX680587 | KX680977 | KX620688** | China: Hebei |
| 30872 | *Chaitophorus populeti* | *Populus* sp. | KX680213 | KX680588 | KX680978 | KX681336 | China: Hebei |
| 30894 | *Chaitophorus saliniger* | *Salix* sp. | KX680214 | - | KX680979 | KX681337 | China: Hebei |
| 30946 | *Chaitophorus populeti* | *Populus* sp. | KX680215 | KX680589 | KX680980 | KX681338 | China: Hebei |
| 30969 | *Periphyllus kuwanaii* | *Acer* sp. | KX680216 | KX680590 | KX680981 | KX681339 | China: Beijing |
| 30983 | *Periphyllus acericola* | *Acer heptalobum* | KX680217 | KX680591 | KX680982 | KX681340 | China: Beijing |
| 30989 | *Chaitophorus leucomelas* | *Populus* sp. | KX680218 | KX680592 | KX680983 | KX681341 | China: Beijing |
| 31044 | *Periphyllus koelreuteriae* | *Koelreuteria paniculata* | KX680219 | KX680593 | KX680984 | KX681342 | China: Beijing |
| 31086 | *Chaitophorus populeti* | *Populus* sp. | KX680220 | KX680594 | KX680985 | KX681343 | China: Hebei |
| 31113 | *Periphyllus bulgaricus* | *Acer mono* | KX680221 | KX680595 | KX680986 | KX681344 | China: Hebei |
| 31119 | *Chaitophorus leucomelas* | *Populus* sp. | KX620527** | KX680596 | KX680987 | KX620674** | China: Hebei |
| 31213 | *Chaitophorus leucomelas* | *Populus* sp. | KX620528** | KX680597 | KX680988 | KX620675** | China: Sichuan |
| 31349 | *Chaitophorus saliniger* | *Salix babylonica* | KT237854* | - | KX680989 | KT237267** | China: Sichuan |
| 31466 | *Chaitophorus saliniger* | *Salix* sp. | KT237545* | - | KX680990 | KT236958** | China: Gansu |
| 31489 | *Chaitophorus populeti* | *Populus* sp. | KX680222 | KX680598 | KX680991 | KX681345 | China: Gansu |
| 31565 | *Chaitophorus* sp.9 | *Populus cathayana* | KX680223 | KX680599 | KX680992 | KX681346 | China: Gansu |
| 31580 | *Chaitophorus saliniger* | *Salix babylonica* | KT237546* | - | KX680993 | KT236959** | China: Gansu |
| 31589 | *Chaitophorus* sp.6 | *Populus* sp. | KX620542** | KX680600 | KX680994 | KX620689** | China: Gansu |
| 31622 | *Chaitophorus* sp.9 | *Populus* sp. | KX680224 | KX680601 | KX680995 | KX681347 | China: Gansu |
| 31697 | *Chaitophorus saliniger* | *Salix* sp. | KT237835* | - | KX680996 | KT237248** | China: Shaanxi |
| 31722 | *Chaitophorus* sp.9 | *Populus* sp. | KX680225 | KX680602 | KX680997 | KX681348 | China: Shaanxi |
| 31725 | *Chaitophorus nigricantis* | *Salix spodiophylla* | KX620530** | KX680603 | KX680998 | KX620677** | China: Shaanxi |
| 31767 | *Chaitophorus saliniger* | *Salix babylonica* | KT237644* | - | KX680999 | KT237057** | China: Hubei |
| 31819 | *Chaitophorus saliniger* | *Salix* sp. | KT237665* | - | KX681000 | KT237078** | China: Hubei |
| 31846 | *Chaitophorus saliniger* | *Salix* sp. | KT237597* | - | KX681001 | KT237010** | China: Henan |
| 31902 | *Chaitophorus saliniger* | *Salix babylonica* | KT237575* | - | KX681002 | KT236988** | China: Henan |
| 31944 | *Chaitophorus saliniger* | *Salix babylonica* | KT237800* | - | KX681003 | KT237213** | China: Shanxi |
| 31963 | *Chaitophorus populeti* | *Populus* sp. | KX680226 | KX680604 | KX681004 | KX681349 | China: Shanxi |
| 32010 | *Chaitophorus saliniger* | *Salix babylonica* | KT237823* | - | KX681005 | KT237236** | China: Shanxi |
| 32084 | *Chaitophorus saliniger* | *Salix* sp. | KT237563* | - | KX681006 | KT236976** | China: Hebei |
| 32110 | *Chaitophorus saliniger* | *Salix babylonica* | KT237574* | - | KX681007 | KT236987** | China: Hebei |
| 32111 | *Chaitophorus saliniger* | *Salix* sp. | KT237791* | - | KX681008 | KT237204** | China: Shandong |
| 32120 | *Chaitophorus saliniger* | *Salix babylonica* | KT237799* | - | KX681009 | KT237212** | China: Shandong |
| 32175 | *Chaitophorus saliniger* | *Salix matsudana* | KT237737* | - | KX681010 | KT237150** | China: Liaoning |
| 32216 | *Chaitophorus saliniger* | *Salix babylonica* | KT237755* | - | KX681013 | KT237168** | China: Liaoning |
| 32225 | *Chaitophorus saliniger* | *Salix* sp. | KT237668* | - | KX681014 | KT237081** | China: Jilin |
| 32285 | *Chaitophorus nigricantis* | *Salix* sp. | KX680229 | KX680607 | KX681015 | KX681352 | China: Jilin |
| 32298 | *Chaitophorus nigricantis* | *Salix* sp. | KX620531** | KX680608 | KX681016 | KX620678** | China: Jilin |
| 32306 | *Chaitophorus saliniger* | *Salix babylonica* | KT237695* | - | KX681017 | KT237108** | China: Jilin |
| 32352 | *Chaitophorus* sp.1 | *Populus* sp. | KX680230 | KX680609 | KX681018 | KX681353 | Tajikistan |
| 32393 | *Chaitophorus* sp.1 | *Populus* sp. | KX680231 | KX680610 | KX681019 | KX681354 | Tajikistan |
| 32398 | *Chaitophorus* sp.3 | *Populus simonii* | KX680232 | KX680611 | KX681020 | KX681355 | Tajikistan |
| 32410 | *Chaitophorus* sp.3 | *Populus simonii* | KX680233 | KX680612 | KX681021 | KX681356 | Tajikistan |
| 32461 | *Chaitophorus populeti* | *Populus* sp. | KX680234 | KX680613 | KX681022 | KX681357 | China: Beijing |
| 32462 | *Chaitophorus populialbae* | *Populus* sp. | KX680235 | KX680614 | KX681023 | KX681358 | China: Beijing |
| 32463 | *Chaitophorus populeti* | *Populus* sp. | KX680236 | KX680615 | KX681024 | KX681359 | China: Beijing |
| 32670 | *Chaitophorus indicus* | *Populus* sp. | KX680237 | KX680616 | - | KX681360 | China: Xizang |
| 33090 | *Chaitophorus inouyei* | *Populus* sp. | KX620550** | KX680617 | KX681025 | KX620697** | China: Shaanxi |
| 33104 | *Chaitophorus populeti* | *Populus tomentosa* | KX620538** | KX680618 | KX681026 | KX620685** | China: Shaanxi |
| 33134 | *Chaitophorus saliniger* | *Salix* sp. | KT237510* | - | KX681027 | KT236923** | China: Beijing |
| 33139 | *Chaitophorus populeti* | *Populus tomentosa* | KX680238 | KX680619 | KX681028 | KX681361 | China: Beijing |
| 33148 | *Chaitophorus populeti* | *Populus* sp. | KX680239 | KX680620 | KX681029 | KX681362 | China: Beijing |
| 33166 | *Periphyllus kuwanaii* | *Acer mono* | KX680240 | KX680621 | KX681030 | KX681363 | China: Beijing |
| 33171 | *Chaitophorus* sp.9 | *Populus* sp. | KX680241 | KX680622 | KX681031 | KX681364 | China: Beijing |
| 33181 | *Chaitophorus* sp.9 | *Populus* sp. | KX620556** | KX680623 | KX681032 | KX620703** | China: Beijing |
| 33196 | *Periphyllus kuwanaii* | *Acer mono* | KX680242 | KX680624 | KX681033 | KX681365 | China: Beijing |
| 33201 | *Chaitophorus populeti* | *Populus* sp. | KX680243 | KX680625 | KX681034 | KX681366 | China: Beijing |
| 33202 | *Periphyllus diacerivorus* | *Acer mono* | KX620562** | KX680626 | KX681035 | KX681367 | China: Beijing |
| 33213 | *Periphyllus diacerivorus* | *Acer mono* | KX680244 | KX680627 | KX681036 | KX681368 | China: Beijing |
| 33220 | *Periphyllus diacerivorus* | *Acer mono* | KX680245 | KX680628 | KX681037 | KX681369 | China: Beijing |
| 33243 | *Chaitophorus populeti* | *Populus* sp. | KX680246 | KX680629 | KX681038 | KX681370 | China: Beijing |
| 33256 | *Chaitophorus* sp.9 | *Populus* sp. | KX680247 | KX680630 | KX681039 | KX681371 | China: Beijing |
| 33275 | *Chaitophorus populeti* | *Populus* sp. | KX680248 | KX680631 | KX681040 | KX681372 | China: Heilongjiang |
| 33320 | *Chaitophorus saliniger* | *Salix* sp. | KT237630* | - | KX681041 | KT237043** | China: Heilongjiang |
| 33335 | *Chaitophorus nigricantis* | *Salix babylonica* | KX680249 | KX680632 | KX681042 | KX681373 | China: Heilongjiang |
| 33354 | *Chaitophorus saliniger* | *Salix* sp. | KT237758* | - | KX681043 | KT237171** | China: Inner Mongolia |
| 33405 | *Periphyllus diacerivorus* | *Acer* sp. | KX680250 | KX680633 | KX681044 | KX681374 | China: Inner Mongolia |
| 33412 | *Sipha* (*Rungsia*) *burakowskii* | Gramineae | KX680251 | KX680634 | KX681045 | KX681375 | China: Inner Mongolia |
| 33488 | *Chaitophorus saliniger* | *Salix babylonica* | KT237778* | - | KX681046 | KT237191** | China: Inner Mongolia |
| 33928 | *Chaitophorus* sp.9 | *Populus* sp. | KX680252 | KX680635 | KX681047 | KX681376 | China: Qinghai |
| 33936 | *Chaitophorus* sp.9 | *Populus simonii* | KX680253 | KX680636 | KX681048 | KX681377 | China: Qinghai |
| 33963 | *Chaitophorus* sp.9 | *Populus cathayana* | KX680254 | KX680637 | KX681049 | KX681378 | China: Qinghai |
| 33981 | *Chaitophorus* sp.9 | *Populus cathayana* | KX680255 | KX680638 | KX681050 | KX681379 | China: Qinghai |
| 33982 | *Chaitophorus* sp.9 | *Populus cathayana* | KX680256 | KX680639 | KX681051 | KX681380 | China: Qinghai |
| 33986 | *Chaitophorus* sp.9 | *Populus* sp. | KX680257 | - | - | - | China: Qinghai |
| 34028 | *Chaitophorus* sp.9 | *Populus* sp. | KX680258 | KX680640 | KX681052 | KX681381 | China: Qinghai |
| 34045 | *Chaitophorus* sp.9 | *Populus* sp. | KX680259 | KX680641 | KX681053 | KX681382 | China: Qinghai |
| 34053 | *Chaitophorus* sp.9 | Salicaceae | KX680260 | KX680642 | KX681054 | KX681383 | China: Qinghai |
| 34064 | *Chaitophorus* sp.9 | *Populus* sp. | KX680261 | KX680643 | KX681055 | KX681384 | China: Qinghai |
| 34070 | *Chaitophorus* sp.9 | *Populus simonii* | KX680262 | KX680644 | KX681056 | KX681385 | China: Qinghai |
| 34081 | *Chaitophorus* sp.9 | *Populus* sp. | KX680263 | KX680645 | KX681057 | KX681386 | China: Qinghai |
| 34139 | *Chaitophorus* sp.9 | *Populus canadensis* | KX620557** | KX680646 | KX681058 | KX620704** | China: Qinghai |
| 34141 | *Chaitophorus nigricantis* | *Salix* sp. | KX620532** | KX680647 | KX681059 | KX620679** | China: Qinghai |
| 34143 | *Chaitophorus* sp.9 | *Populus canadensis* | KX680264 | KX680648 | KX681060 | KX681387 | China: Qinghai |
| 34178 | *Chaitophorus nigricantis* | *Salix babylonica* | KX680265 | KX680649 | KX681061 | KX681388 | China: Qinghai |
| 34337 | *Periphyllus koelreuteriae* | *Koelreuteria paniculata* | KX680266 | KX680650 | KX681062 | KX681389 | China: Beijing |
| 34347 | *Chaitophorus leucomelas* | *Populus* sp. | KX680267 | KX680651 | KX681063 | KX681390 | China: Beijing |
| 34348 | *Chaitophorus leucomelas* | *Populus tomentosa* | KX680268 | KX680652 | KX681064 | KX681391 | China: Beijing |
| 34578 | *Chaitophorus saliniger* | *Salix babylonica* | KX680269 | - | KX681065 | KX681392 | China: Yunnan |
| 34817 | *Chaitophorus* sp.9 | *Populus* sp. | KX680270 | KX680653 | KX681066 | KX681393 | China: Yunnan |
| 34839 | *Chaitophorus saliniger* | *Salix* sp. | KX680271 | - | KX681067 | KX681394 | China: Yunnan |
| 34862 | *Chaitophorus saliniger* | *Salix* sp. | KX680272 | - | KX681068 | KX681395 | China: Sichuan |
| 34863 | *Chaitophorus saliniger* | *Salix* sp. | KX680273 | - | KX681069 | KX681396 | China: Sichuan |
| 35091 | *Chaitophorus* sp.9 | *Populus davidiana* | KX680274 | KX680654 | KX681070 | KX681397 | China: Heilongjiang |
| 35123 | *Chaitophorus populeti* | *Populus* sp. | KX680275 | KX680655 | KX681071 | - | China: Heilongjiang |
| 35125 | *Chaitophorus populeti* | *Populus* sp. | KX680276 | KX680656 | KX681072 | - | China: Heilongjiang |
| 35126 | *Chaitophorus* sp.8 | *Populus* sp. | KX680277 | KX680657 | KX681073 | KX681398 | China: Heilongjiang |
| 35162 | *Periphyllus acericola* | *Acer* sp. | KX680278 | KX680658 | KX681074 | KX681399 | China: Heilongjiang |
| 35182 | *Chaitophorus capreae* | Salicaceae | KX680279 | KX680659 | KX681075 | KX681400 | China: Heilongjiang |
| 35202 | *Chaitophorus nigricantis* | *Salix* sp. | KX680280 | KX680660 | KX681076 | KX681401 | China: Heilongjiang |
| 35313 | *Chaitophorus indicus* | *Populus* sp. | KX680281 | KX680661 | - | KX681402 | China: Xizang |
| 35314 | *Chaitophorus indicus* | *Populus* sp. | KX680282 | KX680662 | - | KX681403 | China: Xizang |
| 35344 | *Chaitophorus indicus* | *Populus* sp. | KX680283 | KX680663 | - | KX681404 | China: Xizang |
| 35358 | *Chaitophorus kapuri* | *Populus* sp. | KX680284 | KX680664 | KX681077 | KX681405 | China: Xizang |
| 35373 | *Chaitophorus* sp.7 | *Populus* sp. | KX680285 | KX680665 | KX681078 | KX681406 | Tajikistan |
| 35388 | *Chaitophorus saliapterus quinquemaculatus* | *Salix* sp. | KX680286 | KX680666 | KX681079 | KX681407 | Tajikistan |
| 35394 | *Chaitophorus saliapterus quinquemaculatus* | *Salix* sp. | KX680287 | KX680667 | KX681080 | KX681408 | Tajikistan |
| 35402 | *Chaitophorus saliapterus quinquemaculatus* | *Salix* sp. | KX680288 | KX680668 | KX681081 | KX681409 | Tajikistan |
| 35406 | *Chaitophorus saliapterus quinquemaculatus* | *Salix* sp. | KX680289 | KX680669 | KX681082 | KX681410 | Tajikistan |
| 35414 | *Chaitophorus* sp.4 | *Populus* sp. | KX680290 | KX680670 | KX681083 | KX681411 | Tajikistan |
| 35425 | *Chaitophorus saliapterus quinquemaculatus* | *Salix* sp. | KX680291 | KX680671 | KX681084 | KX681412 | Tajikistan |
| 35434 | *Chaitophorus* sp.4 | *Populus* sp. | KX680292 | KX680672 | KX681085 | KX681413 | Tajikistan |
| 35448 | *Chaitophorus* sp.4 | *Populus* sp. | KX680293 | KX680673 | KX681086 | KX681414 | Tajikistan |
| 35455 | *Chaitophorus* sp.3 | *Populus* sp. | KX680294 | KX680674 | KX681087 | KX681415 | Tajikistan |
| 35466 | *Chaitophorus* sp.4 | *Populus* sp. | KX680295 | KX680675 | KX681088 | KX681416 | Tajikistan |
| 35472 | *Chaitophorus nigritus* | *Salix* sp. | KX680296 | KX680676 | KX681089 | KX681417 | Tajikistan |
| 35475 | *Chaitophorus* sp.1 | *Populus* sp. | KX680297 | KX680677 | KX681090 | KX681418 | Tajikistan |
| 35483 | *Chaitophorus saliapterus quinquemaculatus* | *Salix* sp. | KX680298 | KX680678 | KX681091 | KX681419 | Tajikistan |
| 35505 | *Chaitophorus saliniger* | *Salix* sp. | KX680299 | KX680679 | KX681092 | KX681420 | China: Gansu |
| 35510 | *Chaitophorus populeti* | *Populus* sp. | KX680300 | KX680680 | KX681093 | KX681421 | China: Gansu |
| 35523 | *Chaitophorus populeti* | *Populus* sp. | KX680301 | KX680681 | KX681094 | KX681422 | China: Gansu |
| 35536 | *Chaetosiphella stipae* | Gramineae | KX680302 | KX680682 | KX681095 | KX681423 | China: Gansu |
| 35541 | *Chaitophorus populeti* | *Populus* sp. | KX680303 | KX680683 | KX681096 | KX681424 | China: Gansu |
| 35567 | *Chaitophorus populeti* | *Populus* sp. | KX680304 | KX680684 | KX681097 | KX681425 | China: Gansu |
| 35569 | *Chaitophorus populeti* | *Populus* sp. | KX680305 | KX680685 | KX681098 | KX681426 | China: Gansu |
| 35587 | *Chaitophorus saliniger* | *Salix* sp. | KX680306 | - | KX681099 | KX681427 | China: Gansu |
| 35595 | *Chaitophorus* sp.6 | *Populus* sp. | KX680307 | KX680686 | KX681100 | KX681428 | China: Gansu |
| 35647 | *Chaitophorus* sp.9 | *Populus cathayana* | KX680308 | KX680687 | KX681101 | KX681429 | China: Gansu |
| 35664 | *Chaitophorus saliniger* | *Salix* sp. | KX680309 | - | KX681102 | KX681430 | China: Gansu |
| 35723 | *Chaitophorus indicus* | *Populus* sp. | KX680310 | KX680688 | - | KX681431 | China: Xizang |
| 35896 | *Yamatochaitophorus* sp.2 | *Acer* sp. | KX680311 | KX680689 | - | KX681432 | China: Heilongjiang |
| 35983 | *Trichaitophorus* sp. | *Acer mono* | KX680312 | KX680690 | - | KX681433 | China: Xizang |
| 36227 | *Yamatochaitophorus* sp.1 | *Acer* sp. | KX680313 | KX680691 | - | KX681434 | China: Jilin |
| 36254 | *Yamatochaitophorus albus* | *Acer* sp. | KX680314 | KX680692 | - | KX681435 | China: Jilin |
| 36303 | *Chaitophorus populeti* | *Populus* sp. | KX680315 | KX680693 | KX681103 | KX681436 | China: Ningxia |
| 36305 | *Chaitophorus saliniger* | *Salix* sp. | KX680316 | - | KX681104 | KX681437 | China: Ningxia |
| 36317 | *Chaitophorus* sp.6 | *Populus* sp. | KX680317 | KX680694 | KX681105 | KX681438 | China: Ningxia |
| 36324 | *Chaitophorus saliniger* | *Salix* sp. | KX680318 | - | KX681106 | KX681439 | China: Ningxia |
| 36338 | *Chaitophorus populeti* | *Populus* sp. | KX680319 | KX680695 | KX681107 | - | China: Ningxia |
| 36353 | *Chaitophorus leucomelas* | *Populus* sp. | KX680320 | KX680696 | KX681108 | KX681440 | China: Ningxia |
| 36362 | *Chaitophorus saliniger* | *Salix* sp. | KX680321 | - | KX681109 | KX681441 | China: Ningxia |
| 36363 | *Chaitophorus populialbae* | *Populus* sp. | KX680322 | KX680697 | KX681110 | KX681442 | China: Ningxia |
| 36375 | *Chaitophorus populeti* | *Populus* sp. | KX680323 | KX680698 | KX681111 | KX681443 | China: Ningxia |
| 36376 | *Chaitophorus populialbae* | *Populus* sp. | KX680324 | KX680699 | KX681112 | KX681444 | China: Ningxia |
| 36401 | *Chaitophorus populeti* | *Populus* sp. | KX680325 | KX680700 | KX681113 | KX681445 | China: Ningxia |
| 32198-1 | *Periphyllus* sp.3 | *Acer* sp. | KX680227 | KX680605 | KX681011 | KX681350 | China: Liaoning |
| 32198-2 | *Trichaitophorus ginnalarus* | *Acer* sp. | KX680228 | KX680606 | KX681012 | KX681351 | China: Liaoning |
| Y8904 | *Periphyllus* sp.2 | *Acer palmatum* | KX680326 | KX680701 | KX681114 | KX681446 | China: Zhejiang |

* represents released records uploaded by this group; ** represents records that has not yet been released uploaded by this group; - represents sequence that wasn’t gotten; records without symbol were uploaded for this study.

**NCBI data**

| **species** | **COI/GenBank accession no.** | **COII/GenBank accession no.** |
| --- | --- | --- |
| *Atheroides vallescaldera* | KJ737374.1 |  |
| *Chaitophorus capreae* | KF639276.1 |  |
| *Chaitophorus capreae* | KF639277.1 |  |
| *Chaitophorus leucomelas* | KF639278.1 |  |
| *Chaitophorus leucomelas* | KF639279.1 |  |
| *Chaitophorus leucomelas* | KF639280.1 |  |
| *Chaitophorus leucomelas* | KF639281.1 |  |
| *Chaitophorus leucomelas* | KF639282.1 |  |
| *Chaitophorus leucomelas* | KF639283.1 |  |
| *Chaitophorus leucomelas* | KF639284.1 |  |
| *Chaitophorus leucomelas* | KF639285.1 |  |
| *Chaitophorus leucomelas* | KF639286.1 |  |
| *Chaitophorus leucomelas* | KF639287.1 |  |
| *Chaitophorus leucomelas* | KF639288.1 |  |
| *Chaitophorus leucomelas* | KF639289.1 |  |
| *Chaitophorus macrostachyae* | KR039810.1 |  |
| *Chaitophorus neglectus* | KR043639.1 |  |
| *Chaitophorus neglectus* | KR044283.1 |  |
| *Chaitophorus neglectus* | KR030992.1 |  |
| *Chaitophorus neglectus* | KR031199.1 |  |
| *Chaitophorus neglectus* | KR040978.1 |  |
| *Chaitophorus neglectus* | KR045070.1 |  |
| *Chaitophorus neglectus* | KR042102.1 |  |
| *Chaitophorus neglectus* | KR036044.1 |  |
| *Chaitophorus neglectus* | KR031625.1 |  |
| *Chaitophorus neglectus* | KR035786.1 |  |
| *Chaitophorus neglectus* | KR030786.1 |  |
| *Chaitophorus neglectus* | KR037491.1 |  |
| *Chaitophorus neglectus* | KR038566.1 |  |
| *Chaitophorus neglectus* | KR034846.1 |  |
| *Chaitophorus neglectus* | KR031614.1 |  |
| *Chaitophorus neglectus* | KR030673.1 |  |
| *Chaitophorus neglectus* | KR040307.1 |  |
| *Chaitophorus neglectus* | KR031472.1 |  |
| *Chaitophorus neglectus* | KR044816.1 |  |
| *Chaitophorus neglectus* | KR044000.1 |  |
| *Chaitophorus neglectus* | KR039183.1 |  |
| *Chaitophorus neglectus* | KR039586.1 |  |
| *Chaitophorus neglectus* | KR044287.1 |  |
| *Chaitophorus neglectus* | KR038230.1 |  |
| *Chaitophorus nigrae* | EU701575.1 |  |
| *Chaitophorus nigrae* | EU701576.1 |  |
| *Chaitophorus nigrae* | KR042856.1 |  |
| *Chaitophorus nigrae* | KR042669.1 |  |
| *Chaitophorus nigrae* | EU701577.1 |  |
| *Chaitophorus nigrae* | KR044424.1 |  |
| *Chaitophorus nigrae* | KR033946.1 |  |
| *Chaitophorus nigrae* | EU701579.1 |  |
| *Chaitophorus nigrae* | KR044794.1 |  |
| *Chaitophorus nigrae* | KR035534.1 |  |
| *Chaitophorus nigrae* | KR042348.1 |  |
| *Chaitophorus nigrae* | KR043748.1 |  |
| *Chaitophorus nigrae* | KR040181.1 |  |
| *Chaitophorus nigrae* | KR040816.1 |  |
| *Chaitophorus nigrae* | KR030997.1 |  |
| *Chaitophorus nigrae* | KR044826.1 |  |
| *Chaitophorus nigrae* | KR036121.1 |  |
| *Chaitophorus nigrae* | KR039164.1 |  |
| *Chaitophorus nigrae* | KR038957.1 |  |
| *Chaitophorus nigrae* | KR036931.1 |  |
| *Chaitophorus nigrae* | KR035921.1 |  |
| *Chaitophorus nigrae* | KR035703.1 |  |
| *Chaitophorus nigrae* | KR035060.1 |  |
| *Chaitophorus nigrae* | KR032023.1 |  |
| *Chaitophorus nigricantis* | KF639290.1 |  |
| *Chaitophorus nudus* | EU701578.1 |  |
| *Chaitophorus nudus* | KR043560.1 |  |
| *Chaitophorus nudus* | KR041428.1 |  |
| *Chaitophorus pallipes* | KR034133.1 |  |
| *Chaitophorus pallipes* | KR039640.1 |  |
| *Chaitophorus pallipes* | KR037205.1 |  |
| *Chaitophorus pallipes* | KR036201.1 |  |
| *Chaitophorus pallipes* | KR036161.1 |  |
| *Chaitophorus pallipes* | KR033933.1 |  |
| *Chaitophorus populeti* | GU978838.1 |  |
| *Chaitophorus populeti* | KF639291.1 |  |
| *Chaitophorus populeti* | KF639292.1 |  |
| *Chaitophorus populeti* | KF639293.1 |  |
| *Chaitophorus populeti* | KF639294.1 |  |
| *Chaitophorus populeti* | KF639295.1 |  |
| *Chaitophorus populeti* | KF639296.1 |  |
| *Chaitophorus populeti* | KF639297.1 |  |
| *Chaitophorus populeti* | KF639298.1 |  |
| *Chaitophorus populeti* | KF639299.1 |  |
| *Chaitophorus populeti* | KF639300.1 |  |
| *Chaitophorus populeti* | KF311115.1 | KF311139.1 |
| *Chaitophorus populialbae* | KR034300.1 |  |
| *Chaitophorus populialbae* | KF639301.1 |  |
| *Chaitophorus populialbae* | KF639302.1 |  |
| *Chaitophorus populialbae* | KF639303.1 |  |
| *Chaitophorus populialbae* | KF639304.1 |  |
| *Chaitophorus populicola* | EU701592.1 |  |
| *Chaitophorus populicola* | EU701584.1 |  |
| *Chaitophorus populicola* | KR045258.1 |  |
| *Chaitophorus populicola* | KR033855.1 |  |
| *Chaitophorus populicola* | EU701589.1 |  |
| *Chaitophorus populicola* | KR032024.1 |  |
| *Chaitophorus populicola* | EU701598.1 |  |
| *Chaitophorus populicola* | EU701591.1 |  |
| *Chaitophorus populicola* | EU701604.1 |  |
| *Chaitophorus populicola* | EU701582.1 |  |
| *Chaitophorus populicola* | KR033754.1 |  |
| *Chaitophorus populicola* | KR032799.1 |  |
| *Chaitophorus populicola* | KR032467.1 |  |
| *Chaitophorus populicola* | KR034104.1 |  |
| *Chaitophorus populicola* | KR030564.1 |  |
| *Chaitophorus populicola* | KR033971.1 |  |
| *Chaitophorus populicola* | KR040951.1 |  |
| *Chaitophorus populicola* | KR041366.1 |  |
| *Chaitophorus populicola* | KR035802.1 |  |
| *Chaitophorus populicola* | KR035646.1 |  |
| *Chaitophorus populicola* | KR033890.1 |  |
| *Chaitophorus populicola* | KR037478.1 |  |
| *Chaitophorus populicola* | KR035032.1 |  |
| *Chaitophorus populicola* | KR040605.1 |  |
| *Chaitophorus populicola* | KR034181.1 |  |
| *Chaitophorus populicola* | KR034225.1 |  |
| *Chaitophorus populicola* | KR033649.1 |  |
| *Chaitophorus populicola* | KR031640.1 |  |
| *Chaitophorus populicola* | KR041192.1 |  |
| *Chaitophorus populicola* | KR030945.1 |  |
| *Chaitophorus populicola* | KR035791.1 |  |
| *Chaitophorus populicola* | KR039387.1 |  |
| *Chaitophorus populicola* | KR030810.1 |  |
| *Chaitophorus populicola* | KR033879.1 |  |
| *Chaitophorus populicola* | KR031832.1 |  |
| *Chaitophorus populicola* | KR035426.1 |  |
| *Chaitophorus populicola* | KR030532.1 |  |
| *Chaitophorus populicola* | KR038748.1 |  |
| *Chaitophorus populicola* | KR037798.1 |  |
| *Chaitophorus populicola* | KR031968.1 |  |
| *Chaitophorus populicola* | KR045021.1 |  |
| *Chaitophorus populicola* | KR043548.1 |  |
| *Chaitophorus populicola* | KR032421.1 |  |
| *Chaitophorus populicola* | KR033514.1 |  |
| *Chaitophorus populicola* | KR036212.1 |  |
| *Chaitophorus populicola* | KR045219.1 |  |
| *Chaitophorus populicola* | KR043870.1 |  |
| *Chaitophorus populicola* | KR039304.1 |  |
| *Chaitophorus populicola* | KR034624.1 |  |
| *Chaitophorus populicola* | KR042805.1 |  |
| *Chaitophorus populicola* | KR037597.1 |  |
| *Chaitophorus populicola* | KR039007.1 |  |
| *Chaitophorus populicola* | KR037547.1 |  |
| *Chaitophorus populicola* | KR038587.1 |  |
| *Chaitophorus populifolii* | KR040935.1 |  |
| *Chaitophorus populifolii* | KR036194.1 |  |
| *Chaitophorus populifolii* | EU701605.1 |  |
| *Chaitophorus populifolii* | KR040588.1 |  |
| *Chaitophorus populifolii* | KR032338.1 |  |
| *Chaitophorus populifolii* | KR034287.1 |  |
| *Chaitophorus populifolii* | KR031267.1 |  |
| *Chaitophorus populifolii* | KR041970.1 |  |
| *Chaitophorus populifolii* | KR037737.1 |  |
| *Chaitophorus populifolii* | KR042430.1 |  |
| *Chaitophorus populifolii* | KR039943.1 |  |
| *Chaitophorus populifolii* | KR032270.1 |  |
| *Chaitophorus populifolii* | KR036269.1 |  |
| *Chaitophorus populifolii* | KR033204.1 |  |
| *Chaitophorus populifolii* | KR044759.1 |  |
| *Chaitophorus populifolii* | KR042230.1 |  |
| *Chaitophorus populifolii* | KR035473.1 |  |
| *Chaitophorus populifolii* | KR034765.1 |  |
| *Chaitophorus populifolii* | KR039154.1 |  |
| *Chaitophorus populifolii* | KR043287.1 |  |
| *Chaitophorus populifolii* | KR039318.1 |  |
| *Chaitophorus populifolii* | KR032699.1 |  |
| *Chaitophorus populifolii* | KR035663.1 |  |
| *Chaitophorus populifolii* | KR037447.1 |  |
| *Chaitophorus populifolii* | KR036143.1 |  |
| *Chaitophorus populifolii* | KR033183.1 |  |
| *Chaitophorus populifolii* | KR034024.1 |  |
| *Chaitophorus pusillus* | KR031187.1 |  |
| *Chaitophorus pusillus* | KR043730.1 |  |
| *Chaitophorus pusillus* | KR033737.1 |  |
| *Chaitophorus pusillus* | KR032109.1 |  |
| *Chaitophorus pusillus* | KR043901.1 |  |
| *Chaitophorus pusillus* | KR042375.1 |  |
| *Chaitophorus pustulatus* | KR040109.1 |  |
| *Chaitophorus salicti* | KF639305.1 |  |
| *Chaitophorus salicti* | KF639306.1 |  |
| *Chaitophorus salijaponicus* | KF639307.1 |  |
| *Chaitophorus salijaponicus* | KF639308.1 |  |
| *Chaitophorus salijaponicus* | KF639309.1 |  |
| *Chaitophorus salijaponicus* | KF639310.1 |  |
| *Chaitophorus saliniger* | GU978785.1 |  |
| *Chaitophorus stevensis* | KR031020.1 |  |
| *Chaitophorus stevensis* | KR044805.1 |  |
| *Chaitophorus stevensis* | KR043835.1 |  |
| *Chaitophorus stevensis* | KR034439.1 |  |
| *Chaitophorus stevensis* | KR043960.1 |  |
| *Chaitophorus stevensis* | KR036385.1 |  |
| *Chaitophorus stevensis* | KR043933.1 |  |
| *Chaitophorus stevensis* | KR041737.1 |  |
| *Chaitophorus stevensis* | KR036840.1 |  |
| *Chaitophorus stevensis* | KR040890.1 |  |
| *Chaitophorus stevensis* | KR035485.1 |  |
| *Chaitophorus stevensis* | KR039815.1 |  |
| *Chaitophorus stevensis* | KR039577.1 |  |
| *Chaitophorus stevensis* | KR038226.1 |  |
| *Chaitophorus stevensis* | KR040244.1 |  |
| *Chaitophorus stevensis* | KR036487.1 |  |
| *Chaitophorus truncatus* | KF639311.1 |  |
| *Chaitophorus viminicola* | KR037472.1 |  |
| *Periphyllus acericola* | KF639588.1 |  |
| *Periphyllus allogenes* | GU978811.1 |  |
| *Periphyllus bulgaricus* | KF639589.1 |  |
| *Periphyllus californiensis* | GU978812.1 |  |
| *Periphyllus californiensis* | GU978784.1 |  |
| *Periphyllus koelreuteriae* | GU978834.1 |  |
| *Periphyllus koelreuteriae* | JQ920927.1 |  |
| *Periphyllus koelreuteriae* | KC286720.1 |  |
| *Periphyllus lyropictus* | HQ978930.1 |  |
| *Periphyllus lyropictus* | HQ978931.1 |  |
| *Periphyllus lyropictus* | HQ978932.1 |  |
| *Periphyllus lyropictus* | EU701850.1 |  |
| *Periphyllus lyropictus* | KR034164.1 |  |
| *Periphyllus negundinis* | EU701851.1 |  |
| *Periphyllus negundinis* | KR039582.1 |  |
| *Periphyllus negundinis* | KR041876.1 |  |
| *Periphyllus testudinaceus* | KR039670.1 |  |
| *Periphyllus testudinaceus* | EU701852.1 |  |
| *Periphyllus testudinaceus* | KR034690.1 |  |
| *Periphyllus testudinaceus* | KR038533.1 |  |
| *Periphyllus testudinaceus* | KR038568.1 |  |
| *Periphyllus testudinaceus* | KF639591.1 |  |
| *Periphyllus testudinaceus* | KF639592.1 |  |
| *Periphyllus testudinaceus* | KF639593.1 |  |
| *Periphyllus testudinaceus* | KF639594.1 |  |
| *Periphyllus testudinaceus* | KF639595.1 |  |
| *Periphyllus testudinaceus* | KF639596.1 |  |
| *Periphyllus testudinaceus* | KF639597.1 |  |
| *Periphyllus testudinaceus* | KF639598.1 |  |
| *Sipha elegans* | KR044579.1 |  |
| *Sipha elegans* | KR032958.1 |  |
| *Sipha elegans* | KR031644.1 |  |
| *Sipha elegans* | KF639636.1 |  |
| *Sipha elegans* | KF639637.1 |  |
| *Sipha flava* | KR032698.1 |  |
| *Sipha flava* | KR044391.1 |  |
| *Sipha maydis* | KF639638.1 |  |
| *Sipha maydis* | KF639639.1 |  |
| *Sipha maydis* | KF639640.1 |  |
| *Sipha maydis* | KF639641.1 |  |
| *Sipha maydis* | KF639642.1 |  |
