# Supplementary material for: DNA barcoding and species delimitation of Chaitophorinae (Hemiptera, Aphididae)
Source: Zookeys. 2017 Feb 14;(656):25–50. doi: 10.3897/zookeys.656.11440 (PMC5345361; doi:10.3897/zookeys.656.11440)
Supplement: Supplementary material 2 — Table S2 [file zookeys-656-025-s002.docx]

**Table S2. primers information**

| **molecular marker** | **primers** | **primer sequences(5'-3')** | **references** |
| --- | --- | --- | --- |
| COI | LepF | ATTCAACCAATCATAAAGATATTGG | Foottit et al. (2008) |
|  | LepR | TAAACTTCTGGATGTCCAAAAAATCA | Foottit et al. (2008) |
| COI | HCO2198 | TAAACTTCAGGGTGACCAAAAAATCA | Folmer et al.(1994) |
|  | LCO1490 | GGTCAACAAATCATAAAGATATTGG | Folmer et al.(1994) |
| tRNA/COII | mt2993+ | CATTCATATTCAGAATTACC | Stern (1994) |
|  | A3772 | GAGACCATTACTTGCTTTCAGTCATCT | Normark (1996) |
| cytb | CP1 | GATGATGAAATTTTGGATC | Harry et al. (1998) |
|  | CP2 | CTAATGCAATAACTCCTCC | Harry et al. (1998) |
| gnd | BamHI | CGCGGATCCGGWCCWWSWATWATGCCWGGWGG | Clark et al.(1999) |
|  | ApaI | CGCGGGCCCGTATGWGCWCCAAAATAATCWCKTTGWGCTTG | Clark et al.(1999) |
