# Supplementary material for: DNA barcoding and species delimitation of Chaitophorinae (Hemiptera, Aphididae)
Source: Zookeys. 2017 Feb 14;(656):25–50. doi: 10.3897/zookeys.656.11440 (PMC5345361; doi:10.3897/zookeys.656.11440)
Supplement: Supplementary material 3 — Table S3 [file zookeys-656-025-s003.docx]

**Table S3. The analysis results with ABGD of all datasets**

**COI-670:**

|  | | | | Prior Intraspecific divergence (*p*) | | | | | | | | |
| --- | --- | --- | --- | --- | --- | --- | --- | --- | --- | --- | --- | --- |
| Model | X | Steps | Partition | 0.0352 | 0.0226 | 0.0145 | 0.0093 | 0.0059 | 0.0038 | 0.0024 | 0.0016 | 0.0010 |
| Simple | 1.5 | 10 | Initial | 48 | 60 | 60 | 68 | 68 | 68 | 68 | 303 | 303 |
|  |  |  | Recursive | 56 | 70 | 73 | 77 | 82 | 88 | 91 | 303 | 303 |
| JC69 | 1.5 | 10 | Initial | 48 | 48 | 65 | 70 | 81 | 81 | 81 | 303 | 303 |
|  |  |  | Recursive | 57 | 64 | 74 | 79 | 85 | 88 | 91 | 303 | 303 |
| K2P | 1.5 | 10 | Initial | - | - | 66 | 68 | 81 | 81 | 81 | 303 | 303 |
|  |  |  | Recursive | - | - | 74 | 77 | 85 | 88 | 91 | 303 | 303 |

**COI-338:**

|  | | | | Prior Intraspecific divergence (*p*) | | | | | | |
| --- | --- | --- | --- | --- | --- | --- | --- | --- | --- | --- |
| Model | X | Steps | Partition | 0.0145 | 0.0093 | 0.0059 | 0.0038 | 0.0024 | 0.0016 | 0.0010 |
| Simple | 1.5 | 10 | Initial | 25 | 25 | 46 | 46 | 46 | 133 | 133 |
|  |  |  | Recursive | 36 | 38 | 49 | 50 | 50 | 133 | 133 |
| JC69 | 1.5 | 10 | Initial | 25 | 25 | 46 | 46 | 46 | 133 | 133 |
|  |  |  | Recursive | 36 | 38 | 49 | 50 | 50 | 133 | 133 |
| K2P | 1.5 | 10 | Initial | 20 | 20 | 46 | 46 | 46 | 133 | 133 |
|  |  |  | Recursive | 41 | 42 | 49 | 50 | 50 | 133 | 133 |

**COII-376:**

|  | | | | Prior Intraspecific divergence (*p*) | | | | | | |
| --- | --- | --- | --- | --- | --- | --- | --- | --- | --- | --- |
| Model | X | Steps | Partition | 0.0145 | 0.0093 | 0.0059 | 0.0038 | 0.0024 | 0.0016 | 0.0010 |
| Simple | 1.5 | 10 | Initial | 25 | 25 | 46 | 46 | 46 | 133 | 133 |
|  |  |  | Recursive | 36 | 38 | 49 | 50 | 50 | 133 | 133 |
| JC69 | 1.5 | 10 | Initial | 25 | 25 | 46 | 46 | 46 | 133 | 133 |
|  |  |  | Recursive | 36 | 38 | 49 | 50 | 50 | 133 | 133 |
| K2P | 1.5 | 10 | Initial | 20 | 20 | 46 | 46 | 46 | 133 | 133 |
|  |  |  | Recursive | 41 | 42 | 49 | 50 | 50 | 133 | 133 |

**COII-338:**

|  | | | | Prior Intraspecific divergence (*p*) | | | | | | | |
| --- | --- | --- | --- | --- | --- | --- | --- | --- | --- | --- | --- |
| Model | X | Steps | Partition | 0.0359 | 0.0215 | 0.0129 | 0.0077 | 0.0046 | 0.0028 | 0.0017 | 0.0010 |
| Simple | 1.5 | 10 | Initial | 13 | 43 | 45 | 45 | 45 | 45 | 45 | 114 |
|  |  |  | Recursive | 27 | 43 | 45 | 45 | 45 | 55 | 55 | 114 |
| JC69 | 1.5 | 10 | Initial | 18 | 43 | 43 | 43 | 43 | 43 | 43 | 114 |
|  |  |  | Recursive | 20 | 43 | 43 | 44 | 45 | 55 | 55 | 114 |
| K2P | 1.5 | 10 | Initial | - | 43 | 43 | 43 | 43 | 43 | 43 | 114 |
|  |  |  | Recursive | - | 43 | 43 | 44 | 45 | 55 | 55 | 114 |

**Cytb-413:**

|  | | | | Prior Intraspecific divergence (*p*) | | | | | | | |
| --- | --- | --- | --- | --- | --- | --- | --- | --- | --- | --- | --- |
| Model | X | Steps | Partition | 0.0359 | 0.0215 | 0.0129 | 0.0077 | 0.0046 | 0.0028 | 0.0017 | 0.0010 |
| Simple | 1.5 | 10 | Initial | 47 | 47 | 47 | 47 | 47 | 47 | 47 | 120 |
|  |  |  | Recursive | 47 | 48 | 48 | 52 | 56 | 74 | 74 | 163 |
| JC69 | 1.5 | 10 | Initial | 46 | 46 | 46 | 46 | 46 | 46 | 46 | 120 |
|  |  |  | Recursive | 46 | 47 | 47 | 51 | 55 | 74 | 74 | 163 |
| K2P | 1.5 | 10 | Initial | 48 | 48 | 48 | 48 | 48 | 48 | 48 | 120 |
|  |  |  | Recursive | 48 | 48 | 48 | 52 | 56 | 75 | 75 | 163 |

**Cytb-338:**

|  | | | | Prior Intraspecific divergence (*p*) | | | | | | | |
| --- | --- | --- | --- | --- | --- | --- | --- | --- | --- | --- | --- |
| Model | X | Steps | Partition | 0.0359 | 0.0215 | 0.0129 | 0.0077 | 0.0046 | 0.0028 | 0.0017 | 0.0010 |
| Simple | 1.5 | 10 | Initial | 42 | 42 | 42 | 42 | 42 | 42 | 42 | 103 |
|  |  |  | Recursive | 42 | 43 | 43 | 47 | 51 | 66 | 66 | 132 |
| JC69 | 1.5 | 10 | Initial | 41 | 41 | 41 | 41 | 41 | 41 | 41 | 103 |
|  |  |  | Recursive | 41 | 42 | 42 | 46 | 50 | 65 | 65 | 132 |
| K2P | 1.5 | 10 | Initial | 41 | 41 | 41 | 41 | 41 | 41 | 41 | 103 |
|  |  |  | Recursive | 41 | 42 | 42 | 46 | 50 | 65 | 65 | 132 |

**gnd-396:**

|  | | | | Prior Intraspecific divergence (*p*) | | | | | | | |
| --- | --- | --- | --- | --- | --- | --- | --- | --- | --- | --- | --- |
| Model | X | Steps | Partition | 0.0359 | 0.0215 | 0.0129 | 0.0077 | 0.0046 | 0.0028 | 0.0017 | 0.0010 |
| Simple | 1.5 | 10 | Initial | 15 | 32 | 38 | 46 | 46 | 46 | 46 | 109 |
|  |  |  | Recursive | 22 | 36 | 42 | 47 | 54 | 55 | 59 | 109 |
| JC69 | 1.5 | 10 | Initial | - | 35 | 35 | 46 | 46 | 46 | 46 | 109 |
|  |  |  | Recursive | - | 37 | 42 | 47 | 54 | 55 | 59 | 109 |
| K2P | 1.5 | 10 | Initial | - | 35 | 35 | 35 | 48 | 48 | 48 | 109 |
|  |  |  | Recursive | - | 37 | 42 | 43 | 54 | 55 | 59 | 109 |

**gnd-338:**

|  | | | | Prior Intraspecific divergence (*p*) | | | | | | | |
| --- | --- | --- | --- | --- | --- | --- | --- | --- | --- | --- | --- |
| Model | X | Steps | Partition | 0.0359 | 0.0215 | 0.0129 | 0.0077 | 0.0046 | 0.0028 | 0.0017 | 0.0010 |
| Simple | 1.5 | 10 | Initial | - | 30 | 35 | 44 | 44 | 44 | 44 | 94 |
|  |  |  | Recursive | - | 33 | 39 | 44 | 47 | 48 | 50 | 94 |
| JC69 | 1.5 | 10 | Initial | - | 32 | 32 | 44 | 44 | 44 | 44 | 94 |
|  |  |  | Recursive | - | 34 | 39 | 44 | 47 | 48 | 50 | 94 |
| K2P | 1.5 | 10 | Initial | - | 32 | 32 | 44 | 44 | 44 | 44 | 94 |
|  |  |  | Recursive | - | 34 | 39 | 44 | 47 | 48 | 50 | 94 |
